# SARS-CoV-2 Attack Rate and Population Immunity in Southern New England, March 2020 to May 2021

**DOI:** 10.1001/jamanetworkopen.2022.14171

**Published:** 2022-05-26

**Authors:** Thu Nguyen-Anh Tran, Nathan B. Wikle, Fuhan Yang, Haider Inam, Scott Leighow, Bethany Gentilesco, Philip Chan, Emmy Albert, Emily R. Strong, Justin R. Pritchard, William P. Hanage, Ephraim M. Hanks, Forrest W. Crawford, Maciej F. Boni

**Affiliations:** 1 Center for Infectious Disease Dynamics, Department of Biology, Pennsylvania State University, University Park; 2 Center for Infectious Disease Dynamics, Department of Statistics, Pennsylvania State University, University Park; 3 Center for Infectious Disease Dynamics, Department of Bioengineering, Pennsylvania State University, University Park; 4 Department of Medicine, Brown University, Providence, Rhode Island; 5 Department of Physics, Pennsylvania State University, University Park; 6 Center for Communicable Disease Dynamics, Department of Epidemiology, Harvard T.H. Chan School of Public Health, Boston, Massachusetts; 7 Department of Biostatistics, Yale School of Public Health, New Haven, Connecticut; 8 Department of Statistics and Data Science, Yale University, New Haven, Connecticut

## Abstract

**Importance:**

In emergency epidemic and pandemic settings, public health agencies need to be able to measure the population-level attack rate, defined as the total percentage of the population infected thus far. During vaccination campaigns in such settings, public health agencies need to be able to assess how much the vaccination campaign is contributing to population immunity; specifically, the proportion of vaccines being administered to individuals who are already seropositive must be estimated.

**Objective:**

To estimate population-level immunity to SARS-CoV-2 through May 31, 2021, in Rhode Island, Massachusetts, and Connecticut.

**Design, Setting, and Participants:**

This observational case series assessed cases, hospitalizations, intensive care unit occupancy, ventilator occupancy, and deaths from March 1, 2020, to May 31, 2021, in Rhode Island, Massachusetts, and Connecticut. Data were analyzed from July 2021 to November 2021.

**Exposures:**

COVID-19–positive test result reported to state department of health.

**Main Outcomes and Measures:**

The main outcomes were statistical estimates, from a bayesian inference framework, of the percentage of individuals as of May 31, 2021, who were (1) previously infected and vaccinated, (2) previously uninfected and vaccinated, and (3) previously infected but not vaccinated.

**Results:**

At the state level, there were a total of 1 160 435 confirmed COVID-19 cases in Rhode Island, Massachusetts, and Connecticut. The median age among individuals with confirmed COVID-19 was 38 years. In autumn 2020, SARS-CoV-2 population immunity (equal to the attack rate at that point) in these states was less than 15%, setting the stage for a large epidemic wave during winter 2020 to 2021. Population immunity estimates for May 31, 2021, were 73.4% (95% credible interval [CrI], 72.9%-74.1%) for Rhode Island, 64.1% (95% CrI, 64.0%-64.4%) for Connecticut, and 66.3% (95% CrI, 65.9%-66.9%) for Massachusetts, indicating that more than 33% of residents in these states were fully susceptible to infection when the Delta variant began spreading in July 2021. Despite high vaccine coverage in these states, population immunity in summer 2021 was lower than planned owing to an estimated 34.1% (95% CrI, 32.9%-35.2%) of vaccines in Rhode Island, 24.6% (95% CrI, 24.3%-25.1%) of vaccines in Connecticut, and 27.6% (95% CrI, 26.8%-28.6%) of vaccines in Massachusetts being distributed to individuals who were already seropositive.

**Conclusions and Relevance:**

These findings suggest that future emergency-setting vaccination planning may have to prioritize high vaccine coverage over optimized vaccine distribution to ensure that sufficient levels of population immunity are reached during the course of an ongoing epidemic or pandemic.

## Key Points

**Question:**

What proportion of individuals living in southern New England had immunity to SARS-CoV-2, either through past infection or vaccination, by May 31, 2021?

**Findings:**

This case series analysis for Rhode Island, Massachusetts, and Connecticut revealed that two-thirds of residents were immune to SARS-CoV-2 by May 31, 2021. The population immune fraction was lower than desired because 27% of vaccines during the winter to spring 2021 vaccination campaign were administered to individuals who were already seropositive.

**Meaning:**

These findings suggest that SARS-CoV-2 population immunity was overestimated in summer 2021 and that future emergency-setting vaccination campaigns may need to exceed traditional coverage goals.

## Teaser

This case series assesses population-level immunity to SARS-CoV-2 during March 2020 through May 2021 in Rhode Island, Massachusetts, and Connecticut.

## Introduction

Public health response and management of the COVID-19 pandemic met significant challenges at every stage of the pandemic in 2020 and 2021. Clinical experience and trial data accrued during the first and most deadly^1,2^ wave of March to April 2020, leading to improvements in care for hospitalized patients.^3,4,5,6^ Understanding of mobility, lockdown, and contact tracing policies improved by the summer of 2020, allowing for preparation of school reopening plans for autumn of 2020.^7,8,9^ However, in autumn of 2020, substantial variation in estimates reported from several large seroprevalence studies^10,11,12^ meant that we knew little at the time about the true number of individuals who had been infected between March 2020 and November 2020 or how population susceptibility would drive the winter epidemic wave of 2020 to 2021.

Real-time estimation of seroprevalence or attack rate is challenging. Model-based estimates of attack rate using daily reported case numbers require us to be able to estimate the number of unreported or untested symptomatic cases and the number of asymptomatic infections. In this estimation procedure, an assumed infection fatality rate (IFR),^13,14^ hospitalization incidence,^1,15^ or death incidence^16^ can be used to work backward to infer the numbers of unreported cases or unreported infections. Alternatively, surveys of health care–seeking behavior can be used.^17,18^ This means that age structure is necessary in these reporting streams, as the rate of asymptomatic SARS-CoV-2 infection, hospitalization probability, and death probability all vary substantially by age.^19,20,21^ When hospitalization incidence is not available (eg, owing to underreporting^1^), data streams for death, current hospitalization, current numbers of patients in intensive care units (ICUs) and using ventilators can be used to estimate the incidence of hospitalization.

Estimating attack rate with cross-sectional serological data presents its own unique requirements, including preplanned periodic serum collections^22,23,24^ and a high-throughput validated assay; results will still be reported with a 1-month lag owing to the delay from infection to immunoglobin G positivity in a serological assay. Since the beginning of the COVID-19 pandemic in the US, the Centers for Disease Control and Prevention (CDC), with a large group of commercial and nonprofit partners,^25,26,27^ has been collecting cross-sectional serum samples from blood donors and residual samples from routine laboratory testing. These sample collections are a valuable epidemiological resource, but for most states, seroprevalence estimates are not translatable into attack rate estimates because the seroprevalence estimates move up and down through time while the attack rate can only go up.^28^ These nonmonotonic measurements are common in serology: if an antibody assay threshold is set too high, the assay shows recent seroprevalence rather than cumulative seroprevalence, resulting in systematic underestimation of the number of individuals who have been infected. A simple example can be seen for Massachusetts infection seroprevalence, measured as 10.2% in late April 2021,^26^ at which point 9.1% of the state’s residents had reported confirmed positive results for SARS-CoV-2 infection. This would mean that the ratio of infections to confirmed cases was 1.1 to 1 in Massachusetts, which is inconsistent with our knowledge of SARS-CoV-2 infection, clinical progression, and reporting. This ratio is typically estimated between 2.0 to 1 and 6.0 to 1, depending on methods and the period being analyzed.^13,14,27^ In this analysis, we present a model-based reconstruction of the SARS-CoV-2 attack rate and population immunity curves for 3 New England states: Massachusetts, Connecticut, and Rhode Island, for the first 15 months of the pandemic.

## Methods

This case series used publicly available population-level count data; all data points were fully deidentified. Studies on publicly available data are exempt from human participants review and informed consent under 45 CFR 46.104 (d)(4)(i).^29^ We used the Strengthening the Reporting of Observational Studies in Epidemiology (STROBE) reporting guideline for clinical observational studies.

A published bayesian inferential framework based on a dynamical epidemic model (eFigure 1 in the Supplement) was used to fit case, hospitalization, and death data from Massachusetts, Connecticut, and Rhode Island.^1,30^ We collected 11 daily data streams from each state: (1) cumulative confirmed cases, (2) cumulative confirmed cases by age, (3) cumulative hospitalized cases, (4) cumulative hospitalized cases by age, (5) number of patients currently hospitalized, (6) number of patients currently in an ICU, (7) number of patients currently receiving mechanical ventilation, (8) cumulative deaths, (9) cumulative deaths by age, (10) cumulative hospital deaths, and (11) cumulative hospital discharges. The age and totals data streams are separated because the age-structured data do not always sum to the correct totals, have more missingness, and require a different statistical approach in the model fitting (eAppendix 1 in the Supplement). Daily time points from March 1, 2020, to June 6, 2021, were included in this analysis. Details on Connecticut data sources are in eAppendix 2 in the Supplement; Rhode Island and Massachusetts data sources are described elsewhere.^1^

### Statistical Analysis

Weekly age-structured SARS-CoV-2 vaccination numbers were obtained separately for the 3 states,^31,32,33,34^ and vaccinated individuals receiving their final vaccine dose were moved from the susceptible compartment in the dynamical model to the recovered compartment whenever a individual who was seronegative was vaccinated. All modeling and data fitting were performed with a daily time step, and weekly vaccination data were configured into a daily time series through simple linear interpolation. The model allows for vaccination of individuals who are seropositive. In the model, the fraction
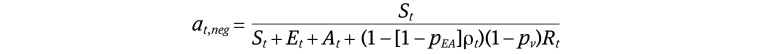
represents the total fraction of nonsymptomatic, nonhospitalized individuals who are antibody-negative and virus-negative at time *t*. The denominator is the candidate pool of individuals for whom a COVID-19 vaccine would be immediately recommended. The uppercase letters represent the numbers of individuals in each model compartment at time *t* for individuals who were susceptible (*S*), exposed individuals (*E*), individuals who were asymptomatically infected with SARS-CoV-2 (*A*), and individuals who recovered from a SARS-CoV-2 infection that did not require hospitalization or those who have been vaccinated (*R*). The fraction *p_ea_* represents the fraction of individuals who were infected but who never developed symptoms (different for every age group), and the reporting rate *ρ_t_* represents the fraction of symptomatic individuals who were tested, confirmed positive, and were thus aware that they had already had COVID-19. The fraction *p_v_* is the total fraction of the population (by age group) that has been vaccinated thus far. Thus, the denominator’s modified *R*-term seeks to approximate the nonvaccinated fraction of the recovered group who were unaware that they have recovered from a SARS-CoV-2 infection, and thus would have sought vaccination between January and May 2021 at the same rate as individuals who had never been infected (only a fraction of these were vaccinated in the model, depending on each state’s data for that week). The model reports the total number of vaccines given to individuals in the susceptible class *S* and the total number of vaccines given to individuals in all classes.

The model accommodates temporally varying patterns of clinical care and changing age-contact rates. To model either a change in clinical management or an increase or decrease in the vulnerability of the current patient pool (ie, the risk of progression to hospitalization or death), we allow the transition probability from medical floor stay to ICU to vary throughout the epidemic.

We report 2 types of IFRs. The population-weighted IFR is the probability of death, if infected, for a person sampled at random from a population with a particular age structure. The epidemic IFR is infections weighted; it is the probability of death for a randomly sampled individual who is infected during a particular epidemic phase. All estimates are presented as medians and 95% credible intervals (CrI) from 1000 posterior samples. Prior distributions are shown in eTable 1 in the Supplement.

Analyses were conducted using the R programming language version 4.1.3 (R Project for Statistical Computing) and the Python programming language version 3.8.12 (Python Software Foundation). Data were analyzed between July 2021 and November 2021.

## Results

A total of 1 160 435 polymerase chain reaction testing–confirmed COVID-19 cases were reported in Massachusetts, Connecticut, and Rhode Island between March 1, 2020, and June 6, 2021. The median age among individuals with confirmed COVID-19 was 38 years, with 19.7% of individuals with COVID-19 older than 60 years and 17.9% of individuals with COVID-19 younger than 20 (eTable 2 in the Supplement). A total of 85 221 individuals with COVID-19 (7.3%) were hospitalized and 28 554 individuals (2.5%) died. In the 3 included states, 6 196 902 individuals, 53.8% of the population, were vaccinated by June 6, 2021.

**Figure 1.**
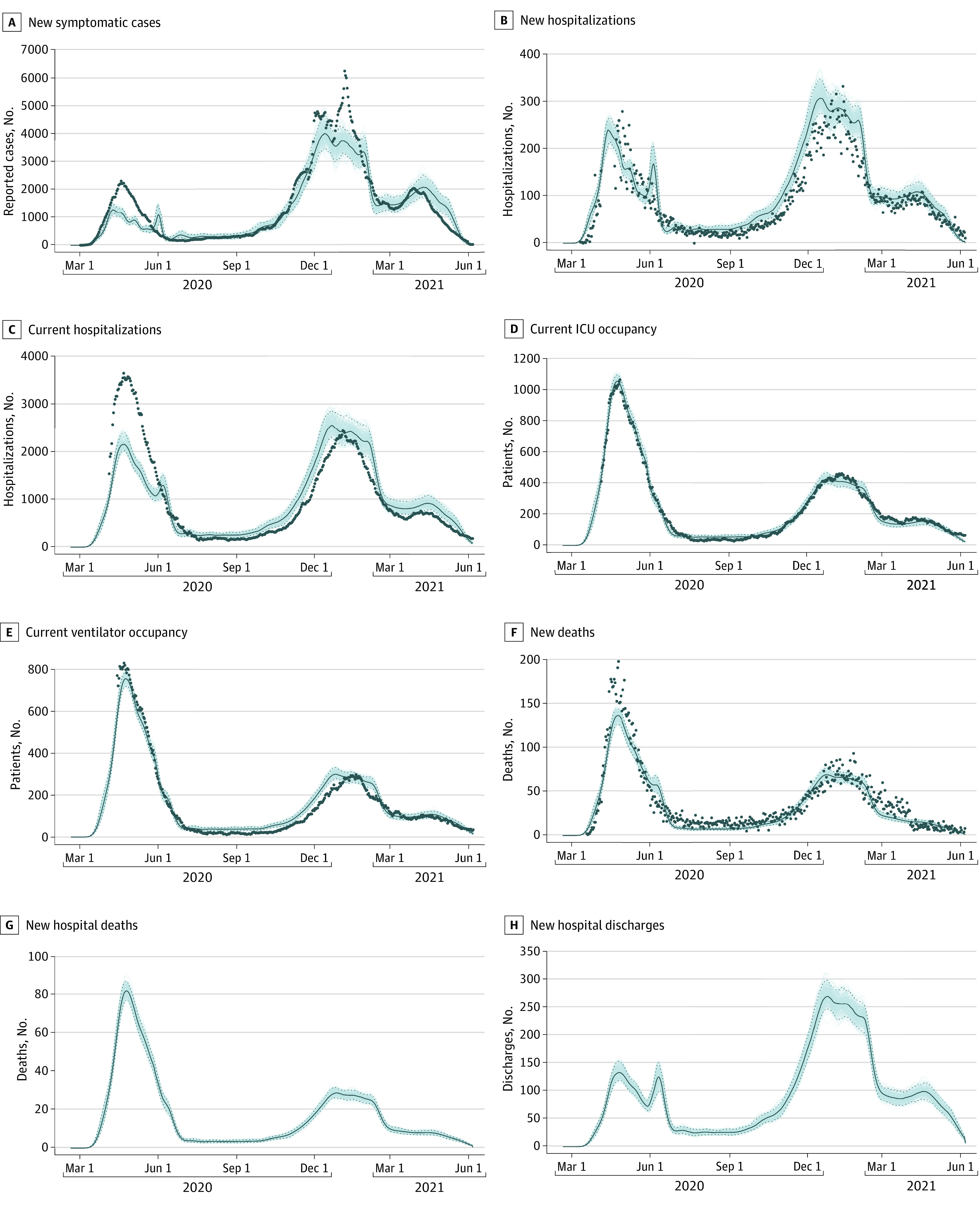
Massachusetts Fit of Model to Data Hospital discharge data and death data separated by in and out of hospital were not available in Massachusetts. Dots indicate absolute daily counts; line, model median from the posterior; and shading, 95% credible region. Similar fits to 11 Rhode Island data streams are shown in eFigure 2 in the Supplement and 7 Connecticut data streams are in eFigure 3 in the Supplement.

State-level inference shows model fits that accurately describe the dynamics of ICU occupancy, ventilator occupancy, and daily death counts in all 3 states during the study period. Rhode Island’s inferred epidemic curve in particular shows close fits to all 8 non–age structured data streams (eFigure 2 in the Supplement), likely owing to the completeness of hospital reporting available in a small state. Case and hospitalization data fit well in all states, with the exception of the case incidence data and current hospitalization data in Massachusetts, which the model underfit for the March to April 2020 epidemic wave (Figure 1). In addition, high variance in new case incidence in Connecticut for the March to April 2020 wave and the major winter wave of 2020 to 2021 suggest that the model may not be capturing complete heterogeneity in transmission dynamics and case reporting (eFigure 3 in the Supplement). Both the data and the model, across all data streams for all 3 states, clearly reconstruct the early epidemic wave of March to April 2020, the summer lull of 2020, the major winter wave of 2020 to 2021, and the lagging wave of the Alpha (B.1.1.7) variant in March to April 2021. All parameter posterior distributions are shown in eFigures 4 through 10 in the Supplement.

**Figure 2.**
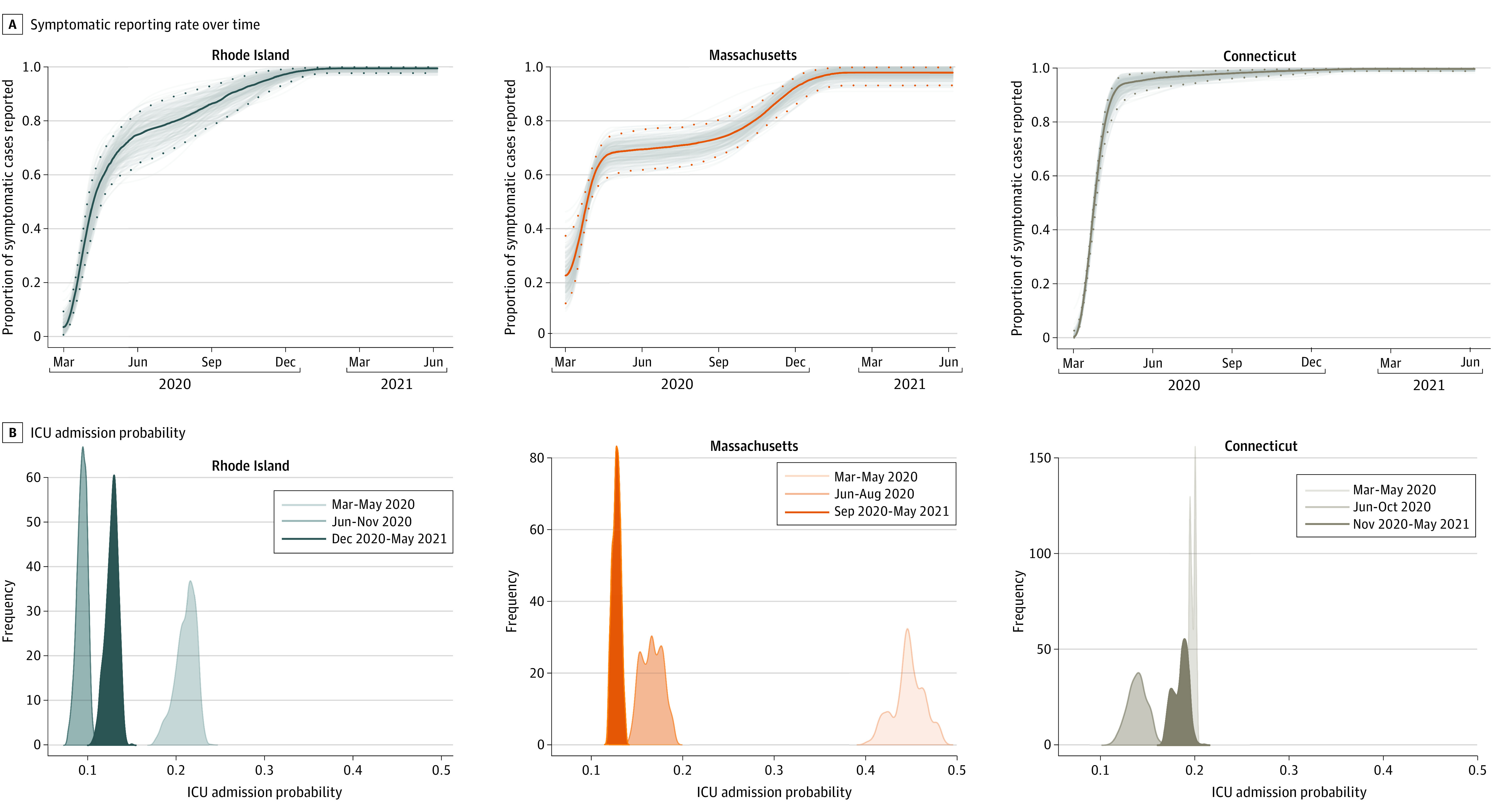
Posterior Distributions for Reporting Rate and Intensive Care Unit (ICU) Admission Probability Posterior distributions for A, the per-symptomatic-case reporting rate, ie, the fraction of symptomatic COVID-19 cases that were reported to the respective Department of Health reporting system, fit with an I-spline to allow an increasing level of testing and reporting through time; and B, ICU admission probability, per hospitalized case, for different phases of the epidemic.

**Figure 3.**
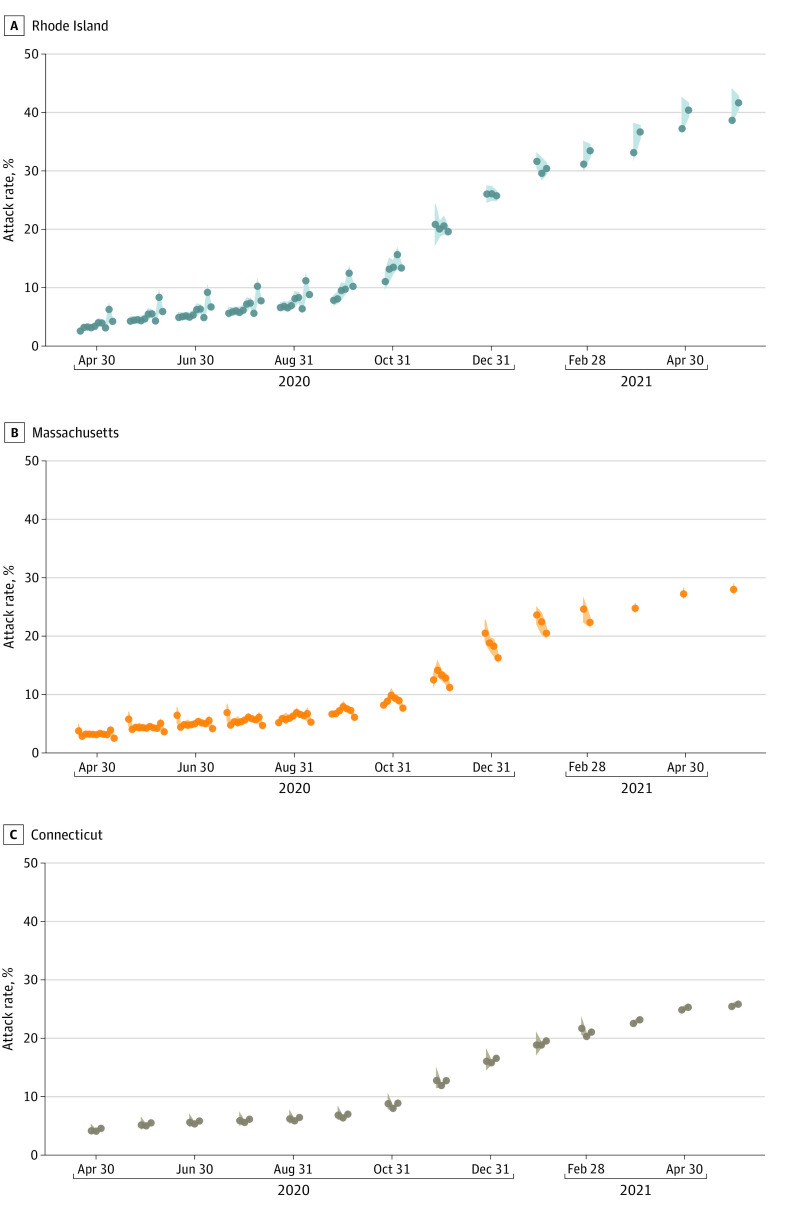
Robustness of Attack Rate Estimation Each dot indicates 1 attack rate estimated with data available only through a particular date. For example, for April 30, 2020, 10 estimates are available for Rhode Island, 11 estimates are available for Massachusetts, and 3 estimates are available for Connecticut; all of these estimates were obtained at different times with different amounts of data available. The dots are ordered from left to right chronologically, with the right-most estimates using the most data (and being done the latest). Shaded areas indicate 95% credible intervals for each estimate.

**Figure 4.**
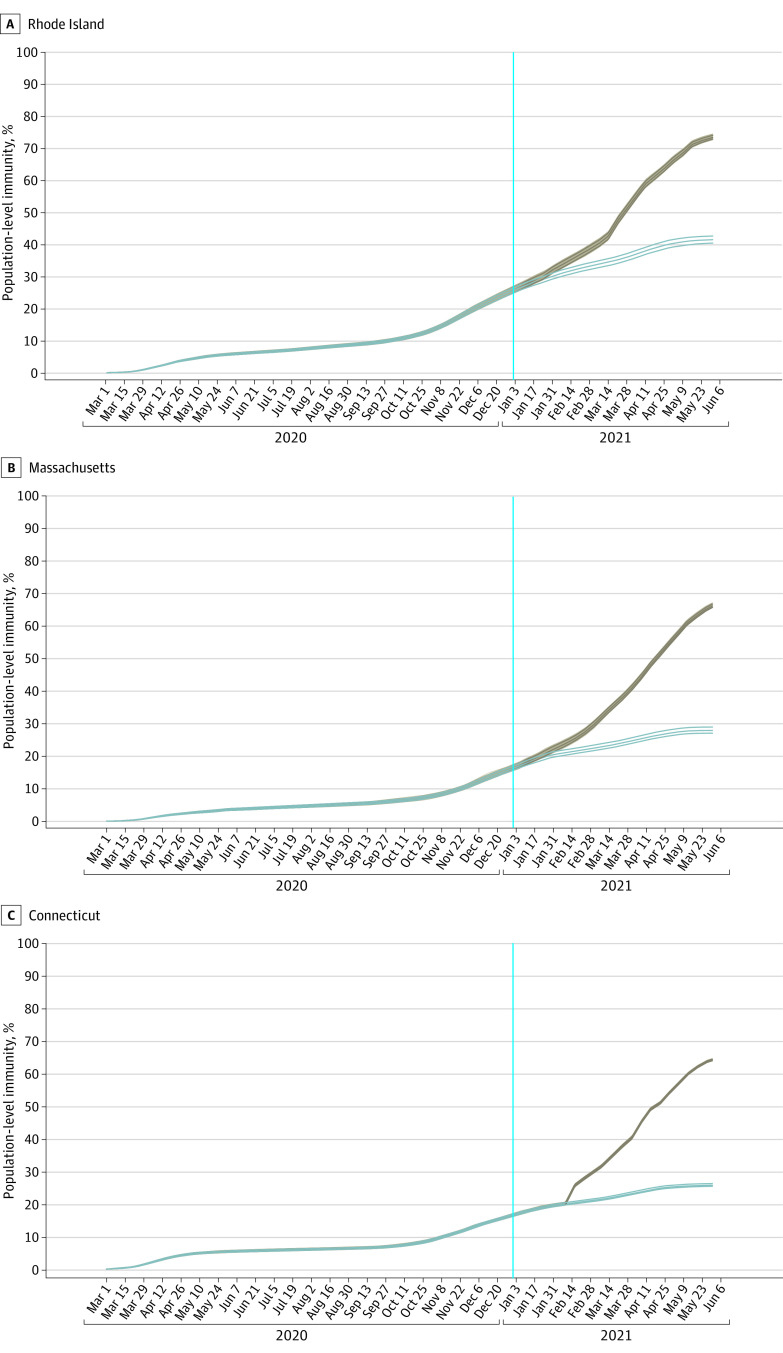
Population Immunity Blue lines indicate total percentage of each state’s population that has been infected; brown lines, percentage of the population that has either been infected or vaccinated (counting only once individuals who have been both infected and vaccinated); vertical line, January 1, 2021. The 3 lines shown are medians and boundaries of 95% credible intervals. Exact estimates are shown in eTables 3 and 4 in the Supplement. Note that the population-level immunity estimate (y-axis) shows the percentage of individuals that have some level of immunity to the Alpha and pre-Alpha variants of SARS-CoV-2.

Using our model’s inferred reporting rate (Figure 2A) and an external estimate^21^ of the asymptomatic fraction of each age group’s infections, we inferred that as of May 31, 2021, the population-level attack rates were 41.5% (95% CrI, 40.4%-42.7%) in Rhode Island, 25.8% (95% CrI, 25.5%-26.3%) in Connecticut, and 28.0% (95% CrI, 27.1%-29.0%) in Massachusetts. Since summer 2020, attack-rate estimations were robust to the differing amounts of data included in the analyses (Figure 3). Attack rate estimates in Connecticut were consistent with those reported in a study by Morozova et al,^35^ and attack rate comparisons in Rhode Island and Massachusetts were consistent with other model-based estimates^13,14^ as described in our previous study.^1^ Comparison with CDC seroprevalence data were more challenging, since there was a discrepancy between the 2 types of estimates presented (eAppendix 3 in the Supplement). By January 31, 2021, approximately 2.1% to 2.3% of each state’s population was vaccinated, with this vaccinated fraction reaching 8.0% to 9.6% by February 28, 2021. Using the modeled number of infections, daily data on vaccinations that were integrated into the model, and the modeled number of individuals who were already seropositive who would have received vaccination, we inferred population immunity levels of 73.4% (95% CrI, 72.9%-74.1%) in Rhode Island, 64.1% (95% CrI, 64.0%-64.4%) in Connecticut, and 66.3% (95% CrI, 65.9%-66.9%) in Massachusetts for May 31, 2021 (Figure 4; eTable 3 and eTable 4 in the Supplement). This implies that more than 33% of southern New England was immunologically naive when the Delta variant reignited a wave of infections in late July 2021. From the model, we infer that the percentage of vaccines administered to individuals who were seropositive was 34.1% (95% CrI, 32.9%-35.2%) in Rhode Island, 24.6% (95% CrI, 24.3%-25.1%) in Connecticut, and 27.6% (95% CrI, 26.8%-28.6%) in Massachusetts. The Table provides a breakdown of infection and vaccination status in all 3 states; these estimates are consistent with those of a study by Moghadas et al^36^ that used a direct IFR-based deaths-to-infections translation to estimate that approximately half of all individuals who had been previously infected received vaccination.

**Table.** Previous Infection Status and Vaccination Status of Included Populations as of May 31, 2021

State	Residents, % (95% credible interval)			
	Previously infected and vaccinated	Vaccinated but not previously infected	Previously infected but not vaccinated	Immunologically naive^a^
Rhode Island	16.5 (16.0-17.1)	31.9 (31.4-32.5)	25.0 (24.4-25.6)	26.6 (25.9-27.1)
Massachusetts	14.7 (14.2-15.2)	38.4 (37.9-38.8)	13.3 (12.8-13.9)	33.7 (33.1-34.1)
Connecticut	12.5 (12.4-12.8)	38.4 (38.1-38.5)	13.2 (13.1-13.5)	35.9 (35.6-36.0)

^a^ No history of infection or vaccination.

The mortality impact of the SARS-CoV-2 epidemic in these 3 states was severe. In Connecticut, 0.229% of residents died during the first 15 months of the epidemic; 0.248% of Rhode Island residents and 0.249% of Massachusetts residents died during this same time period, indicating that the epidemic was approximately 8% to 9% more deadly in Rhode Island and Massachusetts than in Connecticut. Using the inferred attack rates over the first 15 months of the epidemic, we estimate the 15-month epidemic IFR in the 3 states as 0.62% (95% CrI, 0.60%-0.64%) in Rhode Island, 0.89% (95% CrI, 0.87%-0.90%) in Connecticut, and 0.89% (95% CrI, 0.86%-0.92%) in Massachusetts. Rhode Island had an estimated 55% to 60% more infections per population than Connecticut or Massachusetts, and this cannot be explained by any age-specific differences in transmission, indicating that Rhode Island had a larger and broader epidemic across all age groups. The lower epidemic IFR in Rhode Island suggests that the larger epidemic extended to less vulnerable groups (ie, groups less likely to progress to hospitalization and death), lowering the mean fatality rate for the epidemic as a whole.

As in previous analyses showing differing patterns of clinical progression during different epidemic phases,^1,30^ we included changepoints in the ICU admission fraction in our model to allow for changes in clinical treatment for hospitalized patients. In Rhode Island and Massachusetts, the ICU admission fraction dropped substantially from the March to April 2020 epidemic wave to the summer to fall transmission period in 2020; Connecticut estimates were less reliable because ICU data only began to be reported in July 2020. In early summer 2020, the age-adjusted probability of ICU admission decreased from 0.21 (95% CrI, 0.18-0.23) to 0.09 (95% CrI, 0.08-0.10) in Rhode Island, 0.45 (95% CrI, 0.42-0.48) to 0.17 (95% CrI, 0.15-0.19) in Massachusetts, and 0.20 (95% CrI, 0.19-0.20) to 0.14 (95% CrI, 0.12-0.16) in Connecticut (Figure 2B). The estimated population-weighted IFR estimate for the March to May 2020 phase of the epidemic was 1.64% (95% CrI, 1.52%-1.72%) for Rhode Island, 1.55% (95% CrI, 1.45%-1.62%) for Connecticut, and 2.40% (95% CrI, 2.22%-2.51%) for Massachusetts, approximately 2- to 3-fold higher than during later phases of the pandemic, consistent with previous estimates.^1,2^

## Discussion

This case series found that approximately 27% of vaccines in Connecticut, Massachusetts, and Rhode Island were administered to individuals with previous infection, which likely biased the confidence of policy makers and epidemiologists in the vaccination rollout’s ability to generate population immunity and prevent future waves of infection. In general, during the course of the COVID-19 pandemic, an inability to plan several months ahead was partially caused by an inability to quickly and correctly assess the amounts of infection and immunity in the population at a given moment.^37^ The epidemiology community did not foresee the beginning of the Delta wave in July 2021^38^ because there were no accurate state-level estimates of population susceptibility. This had important implications, since approximately 140 000 individuals died in the US during the Delta variant period that lasted from July to October. In addition, we did not have an approach for coanalyzing the waning epidemic dynamics of January to May 2021 with the vaccine rollout that was occurring simultaneously. As a matter of policy for the next emergency-initiated vaccination campaign, it will be necessary to consider excess vaccination as an option to ensure that we are not attempting a so-called soft landing^39,40^ with just enough vaccine distribution to reach an uncertain threshold of population immunity and a slow decline of case rates.

The 3 state epidemic profiles differed. Rhode Island’s epidemic was larger but less severe on a per-infection basis. One potential explanation for Rhode Island’s epidemic profile is a positive correlation between susceptibility and vulnerability. During an epidemic in a heterogeneously exposed population, the most susceptible individuals are infected first.^41,42^ This would mean that in the larger Rhode Island epidemic, the average susceptibility and the average vulnerability would be lower than those in Massachusetts or Connecticut, resulting in fewer hospitalizations per infection and a lower IFR. For Massachusetts, cumulative hospitalization counts are self-reported by hospitals and have not been validated for completeness (Massachusetts Department of Public Health, email and telephone communications, August to October 2020). If hospitalization incidence is undercounted in Massachusetts by 30%, then Massachusetts and Connecticut would have nearly identical epidemic profiles, with 9.4% of the population symptomatically infected, 0.87% of the population hospitalized, and a hospital fatality rate in Massachusetts that is approximately 10% higher than in Connecticut. This could be one of the reasons for the discrepancy between the hospital incidence and current hospitalization data stream in Massachusetts.

The most important information to integrate into the next phase of attack rate estimation and population immunity estimation in the US is the waning rate of SARS-CoV-2 antibodies. Waning antibody rates are now known for the short-term postinfection^43,44,45,46^ and postvaccination periods,^47,48,49,50,51^ suggesting that antibody waning cannot be ignored for SARS-CoV-2 seroprevalence analyses stretching longer than 1 year. Estimates of waning antibodies will allow for the estimation of recent attack rates,^52^ which can either be reported as such or chained together to provide an annual attack rate estimate. Although the initial live integration of these data streams will no doubt be challenging, the benefit will be a situationally aware susceptibility estimate that will allow us to evaluate the invasion ability of a new high-transmissibility variant or immune-escape variant. For Omicron specifically, the reinfection hazards presented by Pulliam et al^53^ indicate that the infected but not vaccinated portion of the population can be viewed as approximately 2-fold as likely to be infected as they were in the previous Delta and Alpha waves. The potential cost of not providing these live attack rate and susceptibility estimates is a repeat of summer 2021, when epidemiologists were caught unaware of the immediate risk posed by the introduction of the Delta variant in a still highly susceptible population.

### Limitations

This study has some limitations. The major limitation in our assessment of the overlap between past infections and vaccination is a lack of data on how individual choices were made to vaccinate or not. Vaccination was not discouraged for individuals with past infection, and most public health communication at the time informed individuals with COVID-19 that they were vaccine-eligible as soon as symptoms resolved. This means that our assumption that individuals with past confirmed SARS-CoV-2 infections would have delayed their vaccinations likely resulted in an underestimate of the vaccine supply that was distributed to individuals with some level of immunity. Additionally, vaccine mandates put in place for high-exposure groups (eg, health care workers, other essential workers, employees working in indoor venues) imply that past exposure and current vaccination should correlate positively. On the other hand, provaccine stances are positively correlated with other types of public health adherence, such as masking or distancing behaviors. This contributes a negative correlative effect between past infection and vaccination. Data to assess the magnitudes of these 3 behavioral associations are not currently available from state departments of health.

## Conclusions

This case series found that ostensibly highly vaccinated populations were susceptible to a surge of Delta infections in July 2021 because we overestimated the spring 2021 vaccination campaign’s effect on population immunity. The real-time exercise organized for the purpose of providing these monthly attack-rate estimates^54^ shows the value of understanding an epidemic’s susceptibility curve while it is changing. Live attack rate estimation can be sharpened by the addition of a data stream that connects case numbers to a whole-population measure. The most direct approach to this is to perform weekly PCR-testing on random samples of the population (or a cohort) to obtain basic live prevalence curves during an epidemic.^55^ Tools like this are resource-intensive but potentially worth the cost; the ability to directly integrate them into sample processing pipelines and data analysis pipelines will hopefully motivate us to include live attack rate and susceptibility estimation into preparation for our next major uncontrolled epidemic or pandemic.

## Notes

Supplement. **eAppendix 1.** Model Description **eFigure 1.** Compartmental Model Diagram **eTable 1.** Priors for Bayesian Inference **eAppendix 2.** Data Streams **eAppendix 3.** Interpretation of Seroprevalence Data
eReferences
**eTable 2.** Demographics of Confirmed COVID-19 Cases **eTable 3.** Attack Rate Estimates from March 2020 to May 2021 **eTable 4.** Population Immunity Estimates from March 2020 to May 2021 **eFigure 2.** Rhode Island Model Fit **eFigure 3.** Connecticut Model Fit **eFigure 4.** Posterior Distributions **eFigure 5.** Posterior Distributions **eFigure 6.** Posterior Distributions **eFigure 7.** Posterior Distributions **eFigure 8.** Posterior Distributions **eFigure 9.** Posterior Distributions **eFigure 10.** Alternate Visualization for Hospitalization Probability
