# Supplementary material for: SARS-CoV-2 Attack Rate and Population Immunity in Southern New England, March 2020 to May 2021
Source: JAMA Netw Open. 2022 May 26;5(5):e2214171. doi: 10.1001/jamanetworkopen.2022.14171 (PMC9136627; doi:10.1001/jamanetworkopen.2022.14171)
Supplement: Supplement. — eAppendix 1. Model Description eFigure 1. Compartmental Model Diagram eTable 1. Priors for Bayesian Inference eAppendix 2. Data Streams eAppendix 3. Interpretation of Seroprevalence Data eReferences eTable 2. Demographics of Confirmed COVID-19 Cases eTable 3. Attack Rate Estimates from March 2020 to May 2021 eTable 4. Population Immunity Estimates from March 2020 to May 2021 eFigure 2. Rhode Island Model Fit eFigure 3. Connecticut Model Fit eFigure 4. Posterior Distributions eFigure 5. Posterior Distributions eFigure 6. Posterior Distributions eFigure 7. Posterior Distributions eFigure 8. Posterior Distributions eFigure 9. Posterior Distributions eFigure 10. Alternate Visualization for Hospitalization Probability [file jamanetwopen-e2214171-s001.pdf]

Supplemental Online Content

Tran TNA, Wikle NB, Yang F, et al. SARS-CoV-2 attack rate and population immunity in southern New England, March 2020 to May 2021. *JAMA Netw Open*. 2022;5(5):e2214171. doi:10.1001/jamanetworkopen.2022.14171

eAppendix 1. Model Description

eFigure 1. Compartmental Model Diagram

eTable 1. Priors for Bayesian Inference

eAppendix 2. Data Streams

eAppendix 3. Interpretation of Seroprevalence Data

eReferences

eTable 2. Demographics of Confirmed COVID-19 Cases

eTable 3. Attack Rate Estimates from March 2020 to May 2021

eTable 4. Population Immunity Estimates from March 2020 to May 2021

eFigure 2. Rhode Island Model Fit

eFigure 3. Connecticut Model Fit

eFigure 4. Posterior Distributions

eFigure 5. Posterior Distributions

eFigure 6. Posterior Distributions

eFigure 7. Posterior Distributions

eFigure 8. Posterior Distributions

eFigure 9. Posterior Distributions

eFigure 10. Alternate Visualization for Hospitalization Probability

This supplemental material has been provided by the authors to give readers additional information about their work.

© 2022 Tran TNA et al. *JAMA Network Open.*

eAppendix 1. Model Description

Full model description and equations are in Section 2 of the Supplementary Materials to Wikle et al[1]. In summary, we use a 30-compartment epidemiological model based on a traditional SEIR framework but with detailed class structure added for the advanced clinical stages of SARS-CoV-2 infection. The model includes twelve types of classes for individuals who are susceptible and immunologically naïve (S), exposed (E), asymptomatic (A), infected and symptomatic (I), acute-phase hospitalized but not in the ICU (H_A), in acute-phase critical care but not yet on a ventilator (C_A), on a ventilator (V), in critical care after removal from mechanical ventilation (C_R), medical-floor hospitalized post-ICU discharge (H_R), recovered from non-hospitalized infection (R), and discharged from hospital upon recovery (R_{HOSP}). Some compartments are broken down in sub-stages. For example, the exposed class is broken down into six sub-classes to reduce the coefficient of variation of the duration of the exposed period from 1.0 to 0.41. The justification for this particular parameter value[2] and all others can be found in the following table

https://github.com/bonilab/covid19-reopening-RI-MA-PA/blob/master/Nov2020/notes/summary.grouped_by_param.md (last accessed March 24 2022)

that summarizes the known studies from 2020 on clinical parameters of duration (how long a particular clinical state lasts for a patient) and probability (likelihood that a patient progresses to a more/less severe clinical state given a current clinical state). As a second example, time-to-death on mechanical ventilation is shorter than time-to-recovery on mechanical ventilation[3–6], and this is reflected in the model structure. eFigure 1 shows the model structure. The model is age-structured, thus every class is broken down into nine sub-compartments corresponding to 10-year age bands.

© 2022 Tran TNA et al. *JAMA Network Open.*

eFigure 1. Compartmental Model Diagram

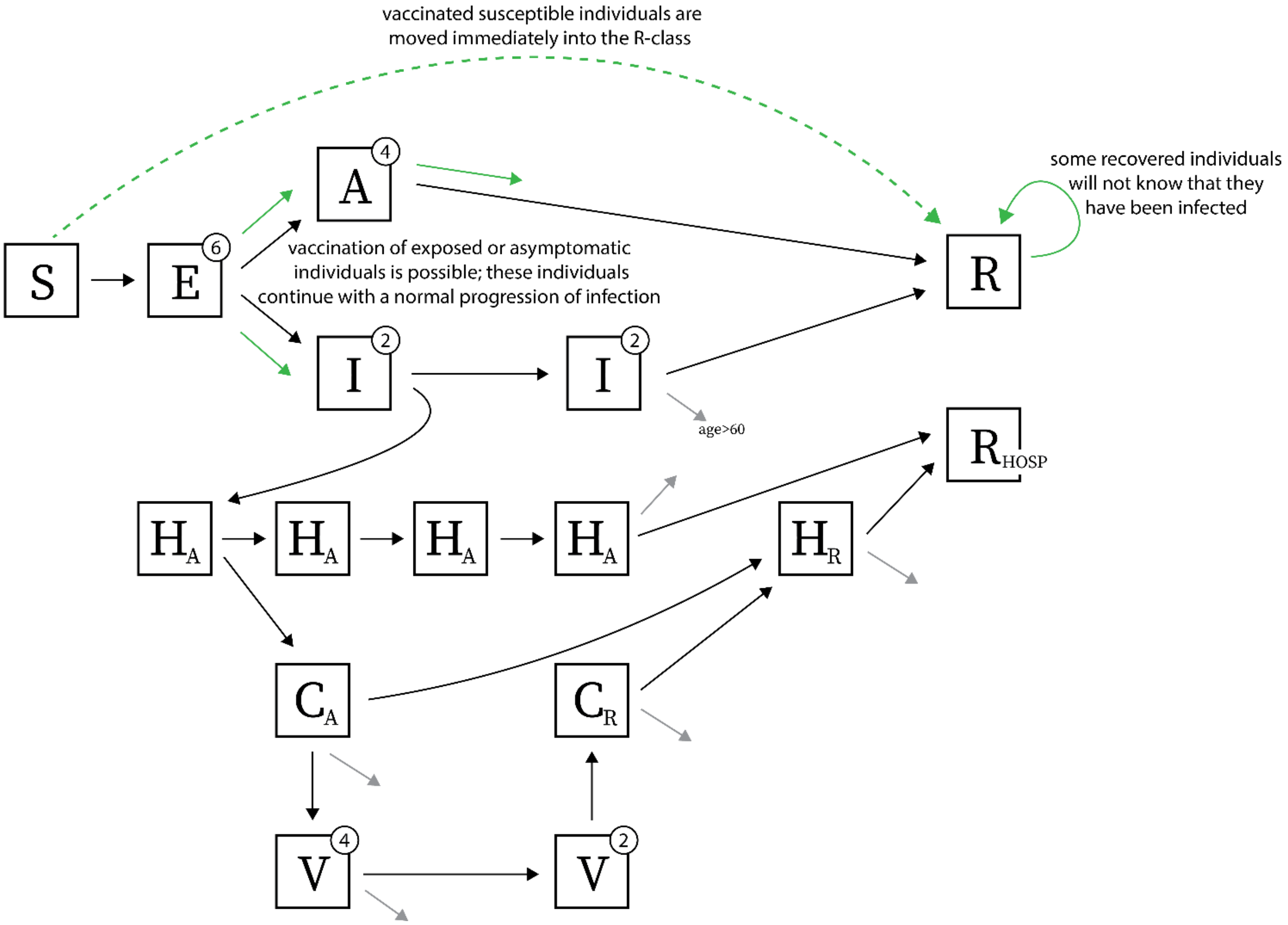

eFigure 1. Compartmental diagram for ordinary differential equations (ODE) epidemic model used for inference. Each compartment is broken down into nine 10-year age bands. Individuals can be susceptible (*S*), exposed (*E*), asymptomatic (*A*), infected and symptomatic but not hospitalized (*I*), hospitalized in the acute stage of infection (H_A), in critical care during acute phase (C_A), on a ventilator (*V*), in critical care after being removed from mechanical ventilation (C_R), hospitalized and convalescing after being discharged from the ICU (H_R), recovered from non-hospitalized infection (*R*), recovered from an infection that required hospitalization (R_{HOSP}). Numbers in upper-right corner of each compartment show number of transitory states used to model that patient group. For example, there are six exposed classes, E_1 to E_6, each lasting approximately one day. Gray arrows indicate death. Green arrows show vaccination, but only the dashed green arrow moves individuals from one state to another.

Vaccination

© 2022 Tran TNA et al. *JAMA Network Open.*

The model is run from March 1 2020 to June 6 2021, and individuals begin to be vaccinated in the model on December 27 2020 in Rhode Island, December 30 2020 in Massachusetts, and February 10 2021 in Connecticut. The starting dates of vaccination campaigns in the model align with dates when the three states started to administer second doses of either the Moderna or Pfizer/BioNTech vaccines. The Connecticut vaccine data did not begin to be recorded until Feb 10 2021 (even though first doses were being distributed prior to January 15, in small numbers), but the CT model fit does not seem to show any irregularities during this early period. State vaccination data are available weekly and are age-structured (some age bands had to be converted to exact 10-year age groups), and these data are transformed into a daily time series through simple linear interpolation. During a model run, once per day, based on each state's data on second doses administered (or a single dose of the Johnson and Johnson vaccine), a number of individuals are chosen to be vaccinated in the model. An individual chosen for vaccination does not necessarily have to be in the S class as some individuals may not know that they were infected in the past or are currently infected; thus, individuals chosen for vaccination can come from any of the S, E, A, or R classes. In early 2021, individuals were encouraged to be vaccinated regardless of past vaccination status, although individuals with known past infections may have delayed their initial vaccinations; this is an assumption we make in our analysis, thus the total number of previously infected individuals that *also* received a SARS-CoV-2 vaccine during Jan-May 2021 represents the a minimum estimate for this overlap. Individuals who are infected but not showing symptoms can also be vaccinated in the model, and they simply continue with their regular course of infection. Equation (1) in the main text shows the fraction of vaccinees that would have been antibody-negative and virus-negative at time of vaccination.

Inference framework and likelihood model

The inference framework is presented in Section 3 of the Supplementary Materials to Wikle et al[1]. Briefly, the likelihood of the data is the product of the likelihood of SARS-CoV-2 incidence data and the likelihood of SARS-CoV-2 prevalence data. Incidence data – symptoms incidence, hospitalization incidence, hospital discharge incidence, and death incidence – are broken down into age groups and totals separately as the

© 2022 Tran TNA et al. *JAMA Network Open.*

sums across age groups do not always add up to the totals. Total incidence is modelled as a partially-observed process (of the differential equations model) with a negative binomial observation function, and age-structured incidence is modeled as a multinomial sample from the total incidence given the model's age-specific incidence trajectories. For days when no age data are available, the multinomial probability component is omitted. Exact likelihood equations are equations (S8) to (S12) in Wikle et al[1].

Current levels of hospitalization, ICU occupancy, and ventilator occupancy are modeled as normal distributions centered on the model's prediction (trajectory) of the number of patients in the hospital, ICU, and on a ventilator that day; the variance of each of these normal distributions is estimated. These normal likelihoods are only added into the full likelihood product every seven days as the three occupancy data streams are strongly auto-correlated.

Priors for parameters

A large number of parameters are estimated in the model, shown in eFigures 4 to 9. A time-varying reporting parameter ρ is estimated (Figure 2A, main text) to show the fraction of symptomatic COVID-19 cases that are captured by the health system (i.e. the proportion of symptomatic infected individuals who choose to seek care, are PCR-tested, and receive a true positive test result that is then sent to the state DOH). The reporting parameter ρ is modeled with an I-spline expansion with breakpoints set at April 1 2020, May 1 2020, July 1 2020, October 14 2020, and December 1 2020 to model an increasing symptomatic reporting rate over time. The spline breakpoints were chosen as we knew from state DOHs that testing access and reporting probability were low in March and possibly April 2020, and the other breakpoints allow for a gradual or rapid increase (depending on the data) of the reporting parameter from summer 2020 to the winter wave of 2020-2021. The reporting rate remains unchanged from December 1 2020 to May 31 2021. Cubic B-spline expansion with one basis function every seven days (total of 66 basis functions) was used to model the population-mixing parameter; see equation (S17) and blue lines in Figure 1 of Wikle et al[1].

© 2022 Tran TNA et al. *JAMA Network Open.*

eTable 1. Priors for Bayesian Inference

Parameter (uniform prior)	**Massachusetts**	**Rhode Island**	**Connecticut**
mean-time-vent (days)	[7.0, 14.0]	[7.0, 14.0]	[7.0, 14.0]
death-prob-home-60	[0.001, 0.2]	[0.001, 0.2]	[0.001, 0.2]
death-prob-home-70	[0.01, 0.3]	[0.01, 0.3]	[0.01, 0.3]
death-prob-home-80	[0.1, 0.4]	[0.1, 0.4]	[0.1, 0.4]
tv-dev-len-hospstay	[0.1, 2.0]	[0.1, 2.0]	[0.1, 2.0]
tv-dev-icu-frac_1	[0.01, 2.0]	[0.01, 1.5]	[0.4, 0.6]
tv-dev-icu-frac_2	[0.01, 2.0]	[0.01, 2.0]	[0.01, 2.0]
tv-dev-icu-frac_3	[0.01, 2.0]	[0.01, 2.0]	[0.01, 2.0]
tv-dev-icu-frac-endday_1	[110, 200] i.e. from April 19 2020 to July 18 2020	[130, 165] i.e. from May 9 2020 to June 13 2020	[140, 210] i.e. from May 19 2020 to July 28 2020
tv-dev-icu-frac-endday_2	[250, 380] i.e. from September 6 2020 to January 14 2021	[180, 360] i.e. from June 28 2020 to December 25 2020	[280, 360] i.e. from October 6 2020 to December 25 2020
prob-icu-vent	[0.4, 1.0]	[0.4, 1.0]	[0.4, 1.0]
dev-ventdeath-mid	[0.4, 1.5]	[0.4, 1.5]	[0.4, 1.5]
tv-hosp-frac-10	[0.001, 0.1]	[0.001, 0.1]	[0.001, 0.1]
tv-hosp-frac-20	[0.001, 0.1]	[0.001, 0.1]	[0.001, 0.1]
tv-hosp-frac-30	[0.005, 0.15]	[0.005, 0.15]	[0.005, 0.15]
tv-hosp-frac-40	[0.005, 0.15]	[0.005, 0.15]	[0.005, 0.15]
tv-hosp-frac-50	[0.01, 0.2]	[0.01, 0.2]	[0.01, 0.2]
tv-hosp-frac-60	[0.05, 0.2]	[0.05, 0.2]	[0.05, 0.2]
tv-hosp-frac-70	[0.1, 0.4]	[0.1, 0.4]	[0.1, 0.4]
tv-hosp-frac-80	[0.1, 0.4]	[0.1, 0.4]	[0.1, 0.4]
First set of age-specific contact rates (8 parameters: tv-contact-rate-10_1, …, tv-contact-rate-80_1)	[0.1, 10.0], identical range for all 8 parameters	[0.1, 10.0], identical range for all 8 parameters	[0.1, 10.0], identical range for all 8 parameters
Second set of age-specific contact rates (8 parameters: tv-contact-rate-10_2, …, tv-contact-rate-80_2)	[0.1, 10.0], identical range for all 8 parameters	[0.1, 10.0], identical range for all 8 parameters	[0.1, 10.0], identical range for all 8 parameters
Third set of age-specific contact rates (8 parameters: tv-contact-	[0.1, 10.0], identical range for all 8 parameters	[0.1, 10.0], identical range for all 8 parameters	[0.1, 10.0], identical range for all 8 parameters

© 2022 Tran TNA et al. *JAMA Network Open.*

rate-10_3, …, tv-contact-rate-80_3)			
Fourth set of age-specific contact rates (8 parameters: tv-contact-rate-10_4, …, tv-contact-rate-80_4)	[0.1, 10.0], identical range for all 8 parameters	NA	NA
tv-contact-rate-endday_1	[100, 190] i.e. from April 9 2020 to July 8 2020	[120, 190] i.e. from April 29 2020 to July 8 2020	[100, 190] i.e. from April 9 2020 to July 8 2020
tv-contact-rate-endday_2	[230, 305] i.e. from August 17 2020 to October 31 2020	[250, 410] i.e. from September 6 2020 to February 13 2021	[250, 390] i.e. from September 6 2020 to January 24 2021
tv-contact-rate-endday_3	[330, 420] i.e. from November 25 2020 to February 23 2021	NA	NA

Priors that differ between states are shown in purple.

The parameter "mean-time-vent" is the mean number of days a surviving patient spends on a ventilator. The parameter "death-prob-home-nn" is the probability that a patient in a particular age class dies of COVID-19 without being hospitalized; these parameters are necessary as positive patients in care in nursing homes were not classified as hospitalized even when infections were severe. Parameters with the word "dev" model a multiplicative deviation from a standard parameter. For example, "tv-dev-len-hospstay" is a scaling factor used to multiply the model's average 10.7 day hospital stay[7] (medical-floor, non-ICU). The "dev-icu-frac" parameters modify the age-specific probabilities of progression to ICU from Lewnard et al[7] (all probabilities are multiplied simultaneously, to keep the relative probabilities the same across ages) and they allow for inference on three separate severity periods during the 15-month epidemic (clinical practice improved during the course of the pandemic). The first period for Connecticut differs from all the others (prior set to [0.4-0.6]) because there were no ICU data for Connecticut until July 15 2020. The two "endday" parameters that follow give the priors for the breakpoints demarking the periods when clinical management in hospital improved; the priors set for these breakpoints are not identical because of the difficulty of fitting these particular parameters (see bottom right panels of eFigure 4). A

© 2022 Tran TNA et al. *JAMA Network Open.*

lower ICU admission fraction suggests that hospitalized patients have improved chances of recovery and a lower chance of death. The first changepoint was inferred as Jun 2 for RI, May 26 for MA, and Jun 5 for CT (medians from posteriors), and the second changepoints were inferred as Dec 10 for RI, Sep 12 for MA, and Nov 6 for CT (see bottom right panels of eFigure 4).

The parameter "`prob-icu-vent`" describes the probability of progressing from ICU care to mechanical ventilation, and the "`dev-ventdeath-mid`" parameter allows for flexible fitting of death probability for the 40-70 age group where uncertainty was the greatest. The "`hosp-frac`" parameters give the age-specific probabilities of progressing from symptomatic infection to hospitalization. Age-specific relative contact rates (or mixing rates) have priors of [0.1, 10.0] where 1.0 is the contact rate for the 0-9 age group (the reference group). Connecticut and Rhode Island have three different periods of age-mixing patterns, while Massachusetts had four periods (based on lower BIC and better visual fit). These three or four periods are separated by the "`endday`" parameters at the bottom of eTable 1, whose priors again are slightly different due to the difficulty of fitting these breakpoints.

Posterior distributions of all parameters that were fit are shown in eFigures 4 to 9.

© 2022 Tran TNA et al. *JAMA Network Open.*

eAppendix 2. Data Streams

Collection of Massachusetts and Rhode Island data streams

Data streams for MA and RI were collected as outlined in Wikle et al[1]. In addition, age-structured cumulative hospitalization incidence was added for MA as this data stream was not available for our previous two analyses[1,8]. Details available in the supplementary materials file available at this link:

https://www.science.org/doi/suppl/10.1126/sciadv.abf9868/suppl_file/sciadv.abf9868_sm.pdf

Aggregation and cleaning of Connecticut data streams

Inference framework for Connecticut was based on the same eleven data streams as for RI and MA: (1) cumulative confirmed cases, (2) cumulative confirmed cases by age, (3) cumulative hospitalized cases, (4) cumulative hospitalized cases by age, (5) number of patients currently hospitalized, (6) number of patients currently in ICU, (7) number of patients currently on mechanical ventilation, (8) cumulative deaths, (9) cumulative deaths by age, (10) cumulative hospital deaths, (11) cumulative hospital discharges. Remember that age-structured data streams (*a*) do not always add up to the totals data stream and (*b*) normally have substantial missingness.

Data streams (1), (2), (5), (8), and (9) were collected from daily test results from the Connecticut Department of Public Health (CT DPH) (https://data.ct.gov/Health-and-Human-Services/COVID-19-daily-and-cumulative-cases-deaths-and-tes/5dch-cm68). Cases were defined as individuals with laboratory-confirmed positive COVID-19 tests. Age-stratified cumulative cases and deaths were obtained from the CT DPH website (https://data.ct.gov/Health-and-Human-Services/COVID-19-Cases-and-Deaths-by-Age-Group/ypz6-8qyf). Currently hospitalized patient counts were collected from the daily reports of CT DPH (https://data.ct.gov/Health-and-Human-Services/COVID-19-Tests-Cases-Hospitalizations-and-Deaths-S/rf3k-f8fg).

The remaining data streams were collected from other sources.

© 2022 Tran TNA et al. *JAMA Network Open.*

Data streams (3) and (4) were obtained from the CDC as there were no available total and age-stratified cumulative hospitalized case numbers available from CT DPH. Specifically, these data streams were calculated from the cumulative hospitalization rates from COVID-NET (https://gis.cdc.gov/grasp/COVIDNet/COVID19_3.html) using the population estimates from 2019 US census (https://www.census.gov/quickfacts/fact/table/US/PST045219). The age groups of cumulative hospitalizations from COVID-NET are 0-4, 5-17, 18-29, 30-39, 40-49, 50-64, 65-74, 75-84, and 85+. These age groups were re-binned to conform with the 10-year age bands used in our analysis. For age <30, we assumed that each age group had the same probability of being hospitalized, thus the age bands were re-binned assuming a uniform distribution of hospitalization in 1-year age bands. For age >50, we assumed that the age distribution of the CT hospitalized population followed the same pattern as Rhode Island. Thus, the hospitalization numbers in CT were re-binned to preserve the relative ratios of hospitalization among the 50-59, 60-69, 70-79, and 80+ age groups.

Data stream (6), the current number of patients in the ICU, was collected from the hospital utilization report from the Department of Health and Human Services (https://healthdata.gov/Hospital/COVID-19-Reported-Patient-Impact-and-Hospital-Capa/g62h-syeh), which includes the number of adult patients currently hospitalized in an ICU beds. These data were available from July 15 2020 forward.

Data stream (7), the number of patients on ventilators, was not available.

Data stream (10), cumulative hospital deaths, was not available.

Data stream (11), cumulative hospital discharges, was available from the COVID Tracking Project (https://covidtracking.com/data/state/connecticut). But this data stream was only available weekly, and only

© 2022 Tran TNA et al. *JAMA Network Open.*

from 6/4/2020 to 10/22/2020, and was thus not used in the data fitting. More recent data (Jan 2022) have this data stream available from 5/1/2020 to 10/22/2020.

Vaccination Data in Connecticut

Total (https://data.ct.gov/Health-and-Human-Services/COVID-19-Vaccination-Status-by-Residence-in-a-SVI-/tttv-egb7) and age-stratified fully vaccinated population numbers (https://data.ct.gov/Health-and-Human-Services/COVID-19-Vaccinations-by-Age-Group/vjim-iz5e) were collected from the CT DPH website. A person is considered fully vaccinated if they received two doses of the Pfizer or Moderna vaccines or one dose of the Johnson & Johnson vaccine.

© 2022 Tran TNA et al. *JAMA Network Open.*

eAppendix 3. Interpretation of Seroprevalence Data

Comparison of our attack-rate estimates to CDC seroprevalence data reveals an important general gap in seroepidemiology in that there is no precise or useful measure of ‘recent seroprevalence’. Antibodies to SARS-CoV-2 wane detectably over a period of 3-7 months, depending on the assay and antigen used[9–13]. For an epidemic analysis over 15 months, it is necessary to account for the effects of antibody waning.

The commercial-lab survey results reported by CDC (https://covid.cdc.gov/covid-data-tracker/#national-lab), using nucleocapsid antigen detection only thus excluding vaccinees, show 6% to 10% seroprevalence in RI, MA, and CT through June 2021. These are much lower than the 26% to 42% estimates we report.

Screenshots below taken March 26 2022 for Massachusetts

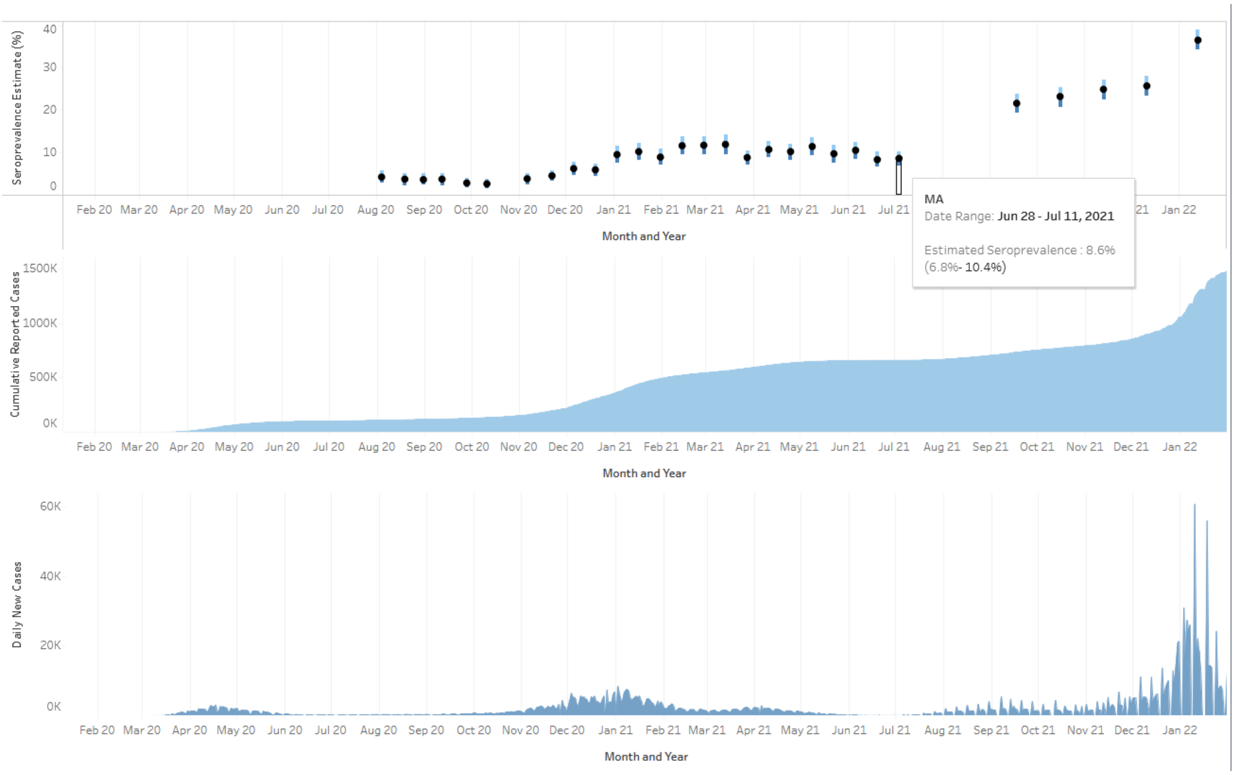

Connecticut

© 2022 Tran TNA et al. *JAMA Network Open.*

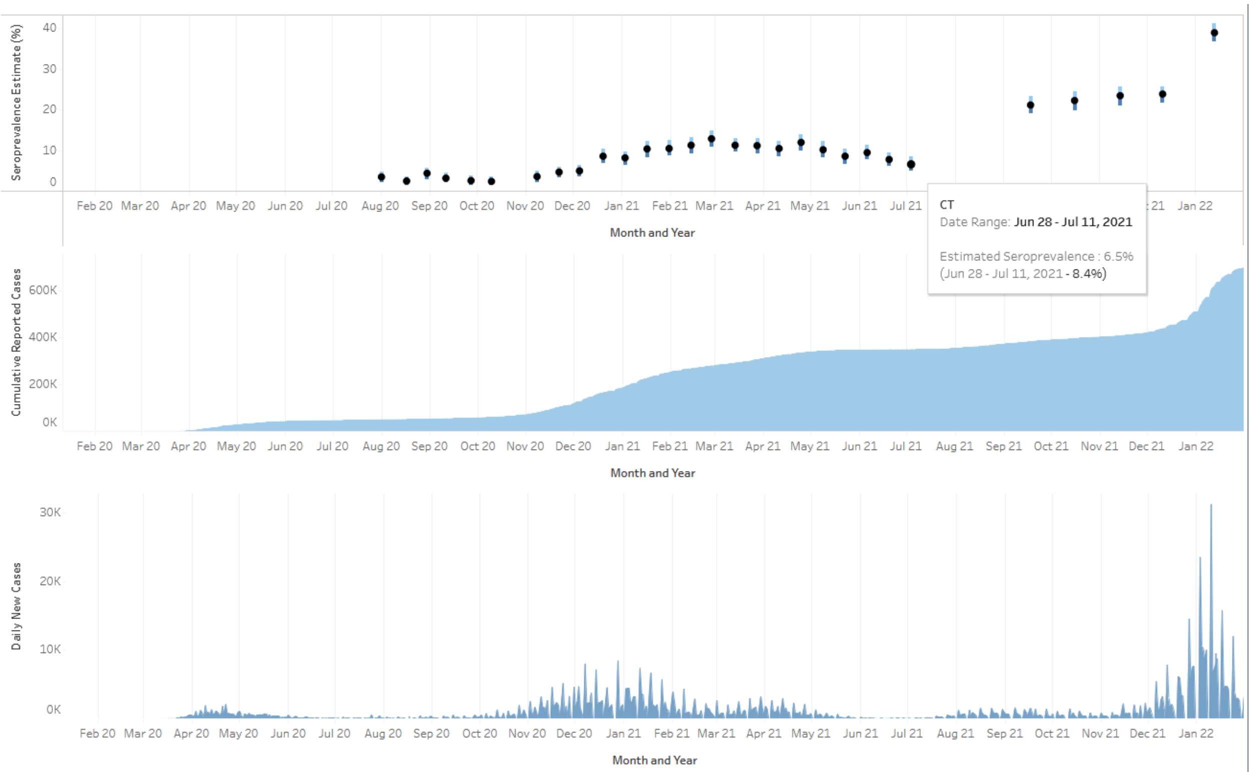

and Rhode Island

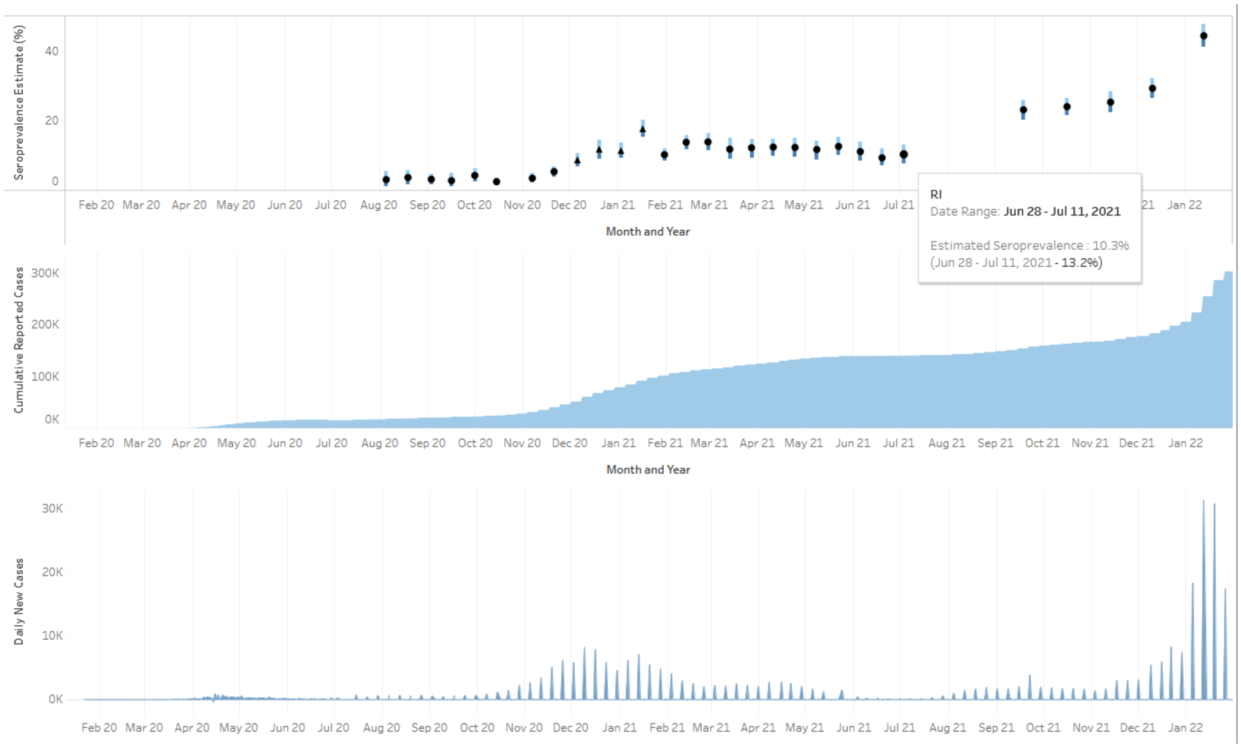

© 2022 Tran TNA et al. *JAMA Network Open.*

The flat/declining seroprevalence estimates from Feb 2021 to June 2021 in these figures show that waning of antibodies was affecting the general-population estimates by introducing a downward bias into what we would want from a cumulative seroprevalence estimate. Each state reported tens of thousands of cases during this period, so the seroprevalence could not possibly have remained constant. The June 2021 estimates on the previous pages are much more accurately described as 'recent attack rate' or 'recent seroincidence', but there is no indication of how much of the recent past this estimate includes.

The CDC blood-donor surveys (https://covid.cdc.gov/covid-data-tracker/#nationwide-blood-donor-seroprevalence) use Spike antigen and thus present an estimate combining past infections and vaccinees. These June 2021 seroprevalence estimates range from 91% to 95% for MA, CT, and RI. The Jones et al[14] paper has May 2021 estimates at >85%.

CDC screenshots below taken March 26 2022, for Massachusetts

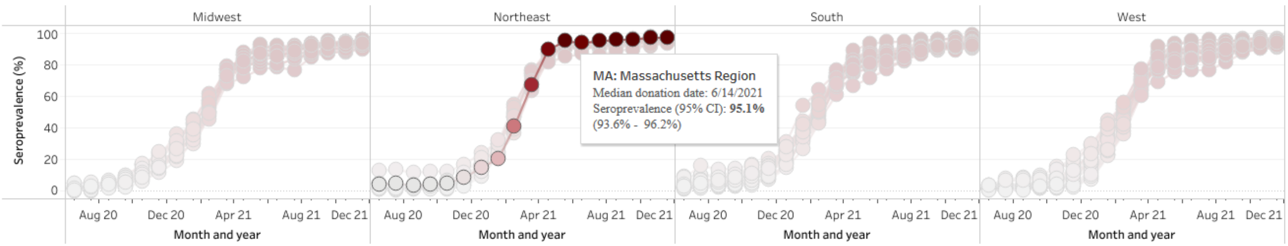

Connecticut

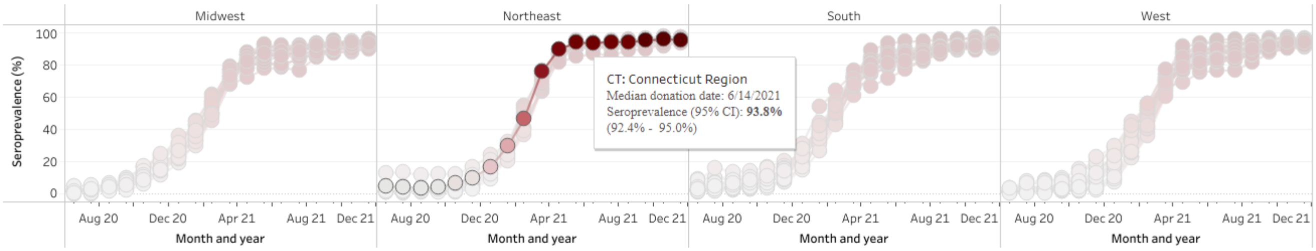

© 2022 Tran TNA et al. *JAMA Network Open.*

and Rhode Island

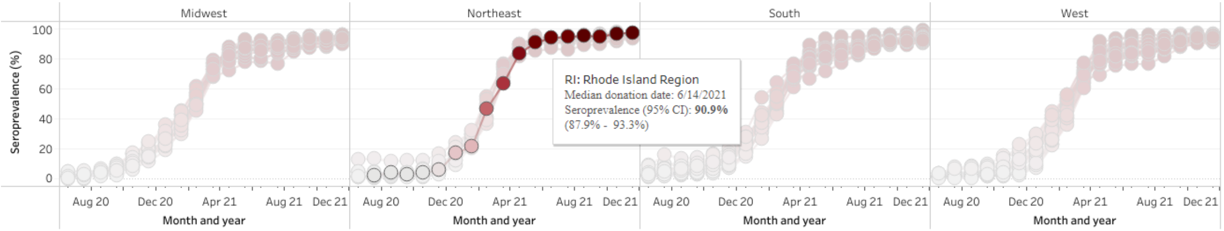

Between 47% and 53% of each state's population was vaccinated by June 2021, thus vaccination numbers cannot make up for the discrepancy between the nucleocapsid (~10%) and Spike (>90%) seroprevalence estimates. It is possible that the Spike estimates are correct, but this is incompatible with our modeling analysis (with estimates between 64% and 73%), other modeling analyses[15,16] that place the infections-to-cases ratio at around 2.0 to 6.0, and the rapid rise of the Delta variant in late July 2021 in New England. The high Spike seroprevalence estimates may be influenced by a positive correlation between being a blood donor and vaccination uptake.

© 2022 Tran TNA et al. *JAMA Network Open.*

eReferences

1. Wikle NB, Tran TNA, Gentilesco B, et al. SARS-CoV-2 epidemic after social and economic reopening in three US states reveals shifts in age structure and clinical characteristics. *Sci Adv*. 2022;8:eabf9868. doi:10.1101/2020.11.17.20232918

2. Lauer SA, Grantz KH, Bi Q, et al. The Incubation Period of Coronavirus Disease 2019 (COVID-19) From Publicly Reported Confirmed Cases: Estimation and Application. *Annals of Internal Medicine*. 2020;172(9):577-582.

3. Gupta S, Hayek SS, Wang W, et al. Factors Associated With Death in Critically Ill Patients With Coronavirus Disease 2019 in the US. *JAMA Intern Med*. 2020;180(11):1436. doi:10.1001/jamainternmed.2020.3596

4. Bhatraju PK, Ghassemieh BJ, Nichols M, et al. Covid-19 in Critically Ill Patients in the Seattle Region — Case Series. *New England Journal of Medicine*. 2020;382(21):2012-2022. doi:10.1056/NEJMoa2004500

5. Cummings MJ, Baldwin MR, Abrams D, et al. Epidemiology, clinical course, and outcomes of critically ill adults with COVID-19 in New York City: a prospective cohort study. *The Lancet*. 2020;395(10239):1763-1770. doi:10.1016/S0140-6736(20)31189-2

6. Ziehr DR, Alladina J, Petri CR, et al. Respiratory Pathophysiology of Mechanically Ventilated Patients with COVID-19: A Cohort Study. *American Journal of Respiratory and Critical Care Medicine*. 2020;201(12):1560-1564. doi:10.1164/rccm.202004-1163LE

7. Lewnard JA, Liu VX, Jackson ML, et al. Incidence, clinical outcomes, and transmission dynamics of severe coronavirus disease 2019 in California and Washington: prospective cohort study. *BMJ*. 2020;369:m1923. doi:10.1136/bmj.m1923

8. Tran TNA, Wikle NB, Albert E, et al. Optimal SARS-CoV-2 vaccine allocation using real-time attack-rate estimates in Rhode Island and Massachusetts. *BMC Med*. 2021;19(1):162. doi:10.1186/s12916-021-02038-w

9. Lau EHY, Tsang OTY, Hui DSC, et al. Neutralizing antibody titres in SARS-CoV-2 infections. *Nat Commun*. 2021;12(1):63. doi:10.1038/s41467-020-20247-4

10. Shioda K, Lau MSY, Kraay ANM, et al. Estimating the Cumulative Incidence of SARS-CoV-2 Infection and the Infection Fatality Ratio in Light of Waning Antibodies. *Epidemiology*. 2021;32(4):518-524. doi:10.1097/EDE.0000000000001361

11. Lumley SF, Wei J, O'Donnell D, et al. *The Duration, Dynamics and Determinants of SARS-CoV-2 Antibody Responses in Individual Healthcare Workers*. Infectious Diseases (except HIV/AIDS); 2020. doi:10.1101/2020.11.02.20224824

12. Ward H, Cooke G, Atchison C, et al. *Declining Prevalence of Antibody Positivity to SARS-CoV-2: A Community Study of 365,000 Adults*.; 2020:2020.10.26.20219725. doi:10.1101/2020.10.26.20219725

13. Peluso MJ, Takahashi S, Hakim J, et al. *SARS-CoV-2 Antibody Magnitude and Detectability Are Driven by Disease Severity, Timing, and Assay*.; 2021:2021.03.03.21251639. doi:10.1101/2021.03.03.21251639

14. Jones JM, Stone M, Sulaeman H, et al. Estimated US Infection- and Vaccine-Induced SARS-CoV-2 Seroprevalence Based on Blood Donations, July 2020-May 2021. *JAMA*. 2021;326(14):1400-1409. doi:10.1001/jama.2021.15161

15. Monod M, Blenkinsop A, Xi X, et al. Age groups that sustain resurging COVID-19 epidemics in the United States. *Science*. 2021;371(6536). doi:10.1126/science.abe8372

16. Unwin HJT, Mishra S, Bradley VC, et al. State-level tracking of COVID-19 in the United States. *Nat Commun*. 2020;11(1):6189. doi:10.1038/s41467-020-19652-6

© 2022 Tran TNA et al. *JAMA Network Open.*

eTable 2. Demographics of Confirmed COVID-19 Cases

age bracket	**Rhode Island**	**Connecticut**	**Massachusetts**
0-9	8344 (5.9%)	20,017 (6.3%)	126,824 (17.9%)
10-19	16,770 (11.8%)	37,685 (11.9%)	
20-29	27,603 (19.4%)	54,631 (17.2%)	133,296 (18.8%)
30-39	22,330 (15.7%)	49,058 (15.4%)	112,906 (15.9%)
40-49	18,712 (13.1%)	44,547 (14.0%)	96.435 (13.6%)
50-59	20,418 (14.3%)	48,075 (15.1%)	101,536 (14.3%)
60-69	14,254 (10.0%)	32,212 (10.1%)	69,170 (9.8%)
70-79	7231 (5.1%)	16,604 (5.2%)	35,407 (5.0%)
80+	6860 (4.8%)	15,085 (4.7%)	33,263 (4.7%)

Numbers (% of total) of COVID-19 cases with age information reported to state DOHs through late May or early June 2021 (June 5 for Rhode Island, June 4 for Connecticut, May 31 for Massachusetts). Massachusetts used a combined 0-19 age bracket.

© 2022 Tran TNA et al. *JAMA Network Open.*

eTable 3. Attack Rate Estimates from March 2020 to May 2021

Date	**Rhode Island**	**Connecticut**	**Massachusetts**
Mar 31 2020	1.17% (1.08% - 1.27%)	1.68% (1.55% - 1.84%)	0.85% (0.76% - 0.94%)
Apr 30 2020	4.04% (3.84% - 4.26%)	4.51% (4.30% - 4.76%)	2.49% (2.27% - 2.71%)
May 31 2020	5.72% (5.45% - 6.00%)	5.45% (5.21% - 5.73%)	3.59% (3.31% - 3.86%)
Jun 30 2020	6.51% (6.23% - 6.83%)	5.77% (5.53% - 6.05%)	4.13% (3.82% - 4.41%)
Jul 31 2020	7.56% (7.25% - 7.91%)	6.07% (5.82% - 6.35%)	4.67% (4.33% - 4.99%)
Aug 31 2020	8.62% (8.28% - 8.99%)	6.37% (6.13% - 6.65%)	5.24% (4.87% - 5.60%)
Sep 30 2020	10.03% (9.65% - 10.44%)	6.94% (6.68% - 7.25%)	6.08% (5.69% - 6.49%)
Oct 31 2020	13.19% (12.71% - 13.74%)	8.82% (8.50% - 9.23%)	7.65% (7.19% - 8.11%)
Nov 30 2020	19.43% (18.77% - 20.13%)	12.67% (12.35% - 13.04%)	11.18% (10.66% - 11.76%)
Dec 31 2020	25.57% (24.74% - 26.41%)	16.50% (16.19% - 16.90%)	16.25% (15.63% - 16.96%)
Jan 31 2021	30.24% (29.29% - 31.19%)	19.48% (19.16% - 19.93%)	20.48% (19.72% - 21.40%)
Feb 28 2021	33.29% (32.28% - 34.37%)	20.99% (20.68% - 21.44%)	22.33% (21.58% - 23.24%)
Mar 31 2021	36.51% (35.42% - 37.64%)	23.18% (22.86% - 23.68%)	24.74% (23.97% - 25.68%)
Apr 30 2021	40.25% (39.14% - 41.48%)	25.24% (24.91% - 25.76%)	27.21% (26.39% - 28.24%)
May 31 2021	41.51% (40.44% - 42.66%)	25.77% (25.45% - 26.29%)	27.96% (27.13% - 29.01%)

Median (95% credible interval) monthly attack rate estimates for Rhode Island, Connecticut, and Massachusetts from March 2020 to May 2021. These are shown as blue lines in Figure 4.

© 2022 Tran TNA et al. *JAMA Network Open.*

eTable 4. Population Immunity Estimates from March 2020 to May 2021

Date	**Rhode Island**	**Connecticut**	**Massachusetts**
Mar 31 2020	1.17% (1.08% - 1.27%)	1.68% (1.55% - 1.84%)	0.85% (0.76% - 0.94%)
Apr 30 2020	4.04% (3.84% - 4.26%)	4.51% (4.30% - 4.76%)	2.49% (2.27% - 2.71%)
May 31 2020	5.72% (5.45% - 6.00%)	5.45% (5.21% - 5.73%)	3.59% (3.31% - 3.86%)
Jun 30 2020	6.51% (6.23% - 6.83%)	5.77% (5.53% - 6.05%)	4.13% (3.82% - 4.41%)
Jul 31 2020	7.56% (7.25% - 7.91%)	6.07% (5.82% - 6.35%)	4.67% (4.33% - 4.99%)
Aug 31 2020	8.62% (8.28% - 8.99%)	6.37% (6.13% - 6.65%)	5.24% (4.87% - 5.60%)
Sep 30 2020	10.03% (9.65% - 10.44%)	6.94% (6.68% - 7.25%)	6.08% (5.69% - 6.49%)
Oct 31 2020	13.19% (12.71% - 13.74%)	8.82% (8.50% - 9.23%)	7.65% (7.19% - 8.11%)
Nov 30 2020	19.43% (18.77% - 20.13%)	12.67% (12.35% - 13.04%)	11.18% (10.66% - 11.76%)
Dec 31 2020	25.57% (24.74% - 26.41%)	16.50% (16.19% - 16.90%)	16.25% (15.63% - 16.96%)
Jan 31 2021	32.21% (31.28% - 33.13%)	19.48% (19.16% - 19.93%)	22.09% (21.34% - 22.99%)
Feb 28 2021	38.70% (37.76% - 39.71%)	29.04% (28.75% - 29.46%)	28.84% (28.14% - 29.69%)
Mar 31 2021	53.28% (52.39% - 54.22%)	40.04% (39.76% - 40.46%)	40.87% (40.21% - 41.66%)
Apr 30 2021	65.78% (65.03% - 66.67%)	54.55% (54.33% - 54.92%)	55.45% (54.86% - 56.17%)
May 31 2021	73.40% (72.89% - 74.08%)	64.13% (63.95% - 64.42%)	66.33% (65.87% - 66.90%)

Median (95% credible interval) monthly population immunity estimates for Rhode Island, Connecticut, and Massachusetts from March 2020 to May 2021. These are shown as green lines in Figure 4.

© 2022 Tran TNA et al. *JAMA Network Open.*

eFigure 2. Rhode Island Model Fit

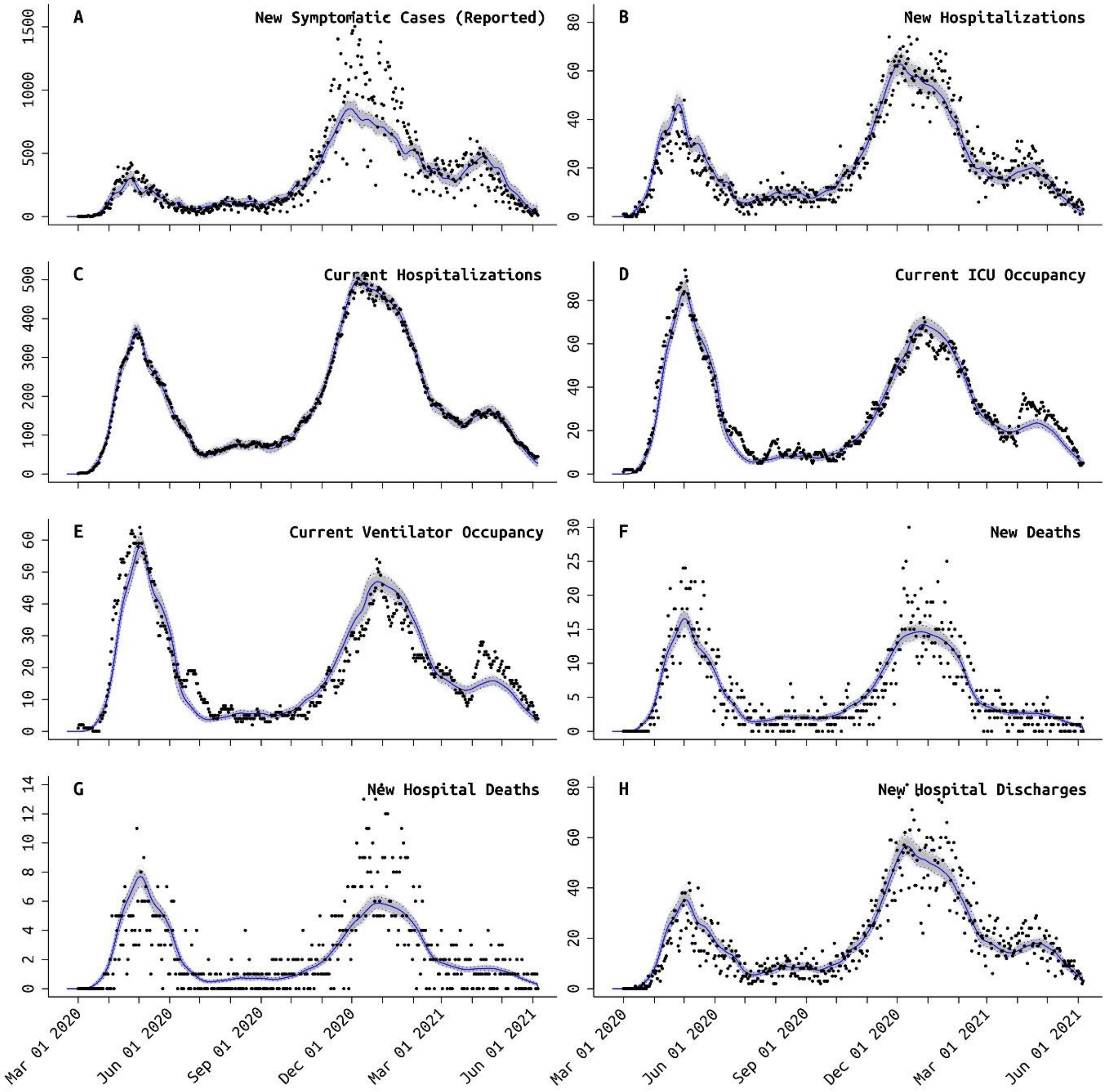

eFigure 2. Rhode Island fit of model to data. Panels **A**, **B**, and **F** also have age-structured data streams, making a total of 11 data streams. Black dots are absolute daily counts. Blue line is model median from the posterior, and gray bands show 95% credible region.

© 2022 Tran TNA et al. *JAMA Network Open.*

eFigure 3. Connecticut Model Fit

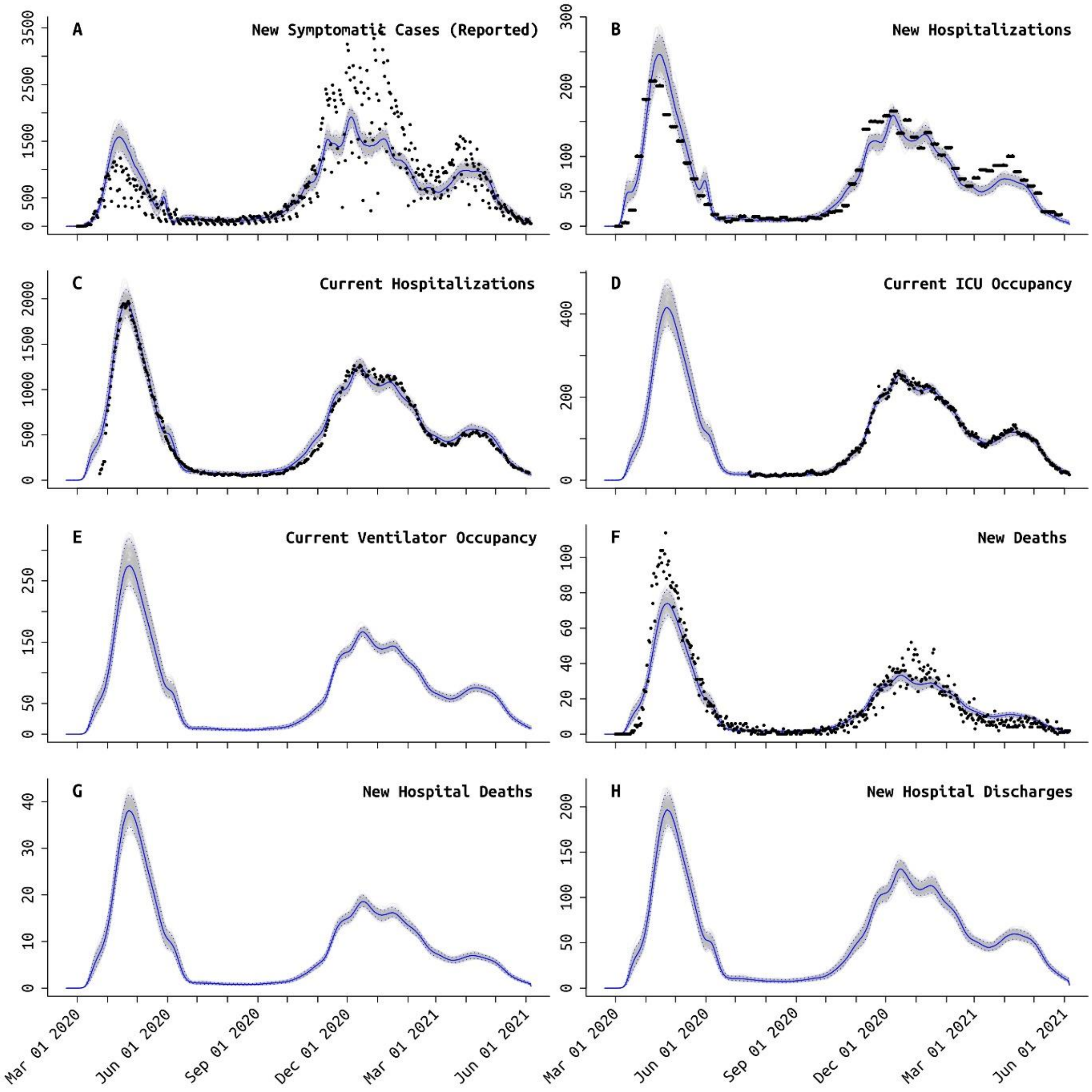

eFigure 3. Connecticut fit of model to data. Panels **A**, **B**, and **F** also have age-structured data streams. Hospital discharge data, death data separated by in/out of hospital, and ventilated patient counts were not available in Connecticut. Black dots are absolute daily counts. Blue line is model median from the posterior, and gray bands show 95% credible region.

© 2022 Tran TNA et al. *JAMA Network Open.*

eFigure 4. Posterior Distributions

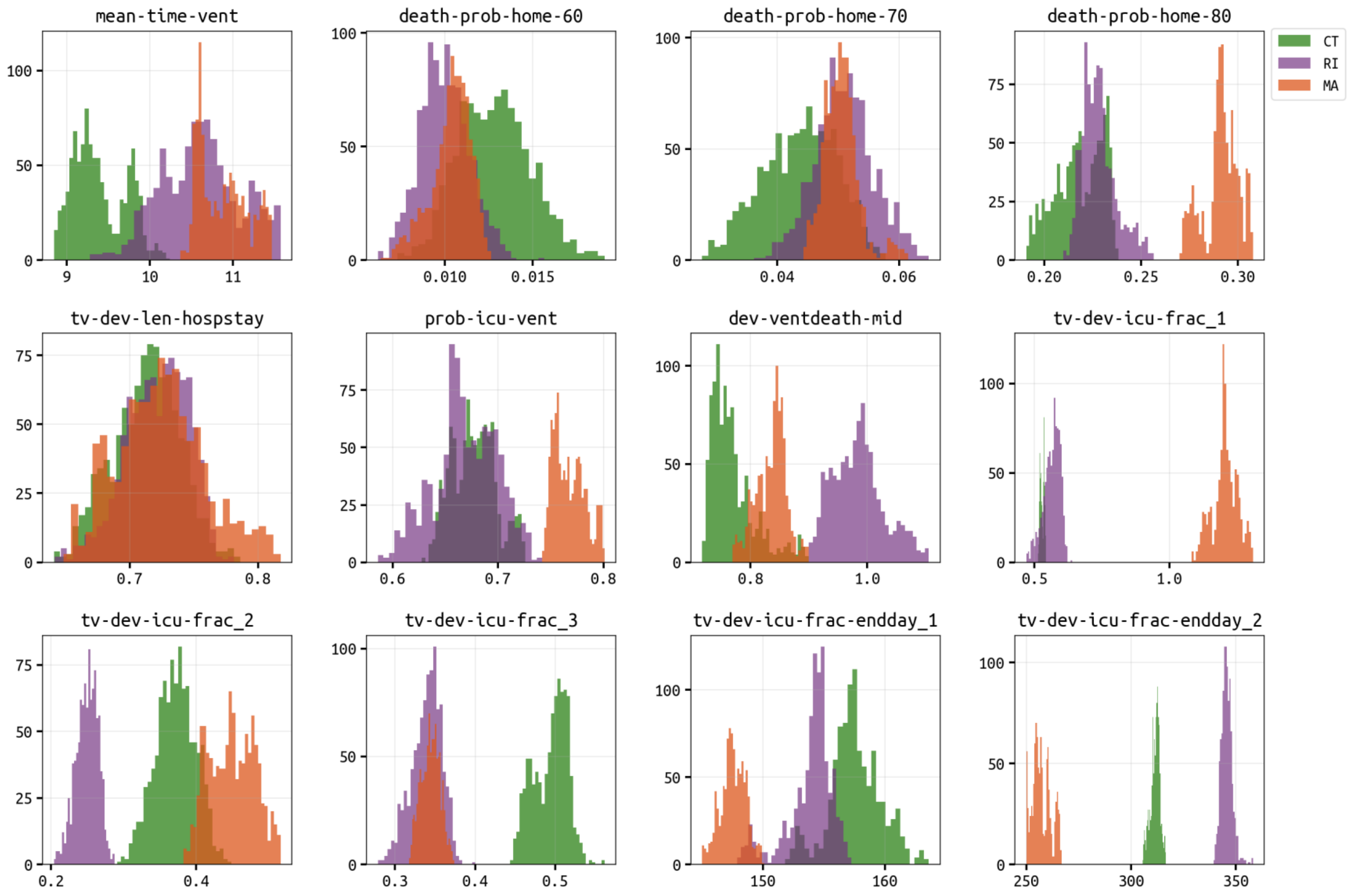

eFigure 4. Panels show posterior distributions for mean number of days a patient spends on a ventilator (**mean-time-vent**); age-specific probability of death in ten-year age bands for symptomatic patients outside hospital settings (e.g. **death-prob-home-60** for the 60-69 age group); the average length of non-ICU hospital stay (**tv-dev-len-hospstay**, multiply by 10.8 days); probability of progression from non-ventilated to ventilated status in the ICU (**prob-icu-vent**); deviation from expected mortality rate for 40-70 year-olds on ventilators (**dev-ventdeath-mid**); for three periods of the epidemic, the relative probability of ICU admission for hospitalized patients (**tv-dev-icu-frac_1**, tv-**dev-icu-frac_2**, **tv-dev-icu-frac_3**); the end-days of the first two periods with aforementioned ICU admission probabilities (**tv-dev-icu-endday_1**, **tv-dev-icu-endday_2**, day 150 is May 30 2020, day 300 is Oct 26 2020).

© 2022 Tran TNA et al. *JAMA Network Open.*

eFigure 5. Posterior Distributions

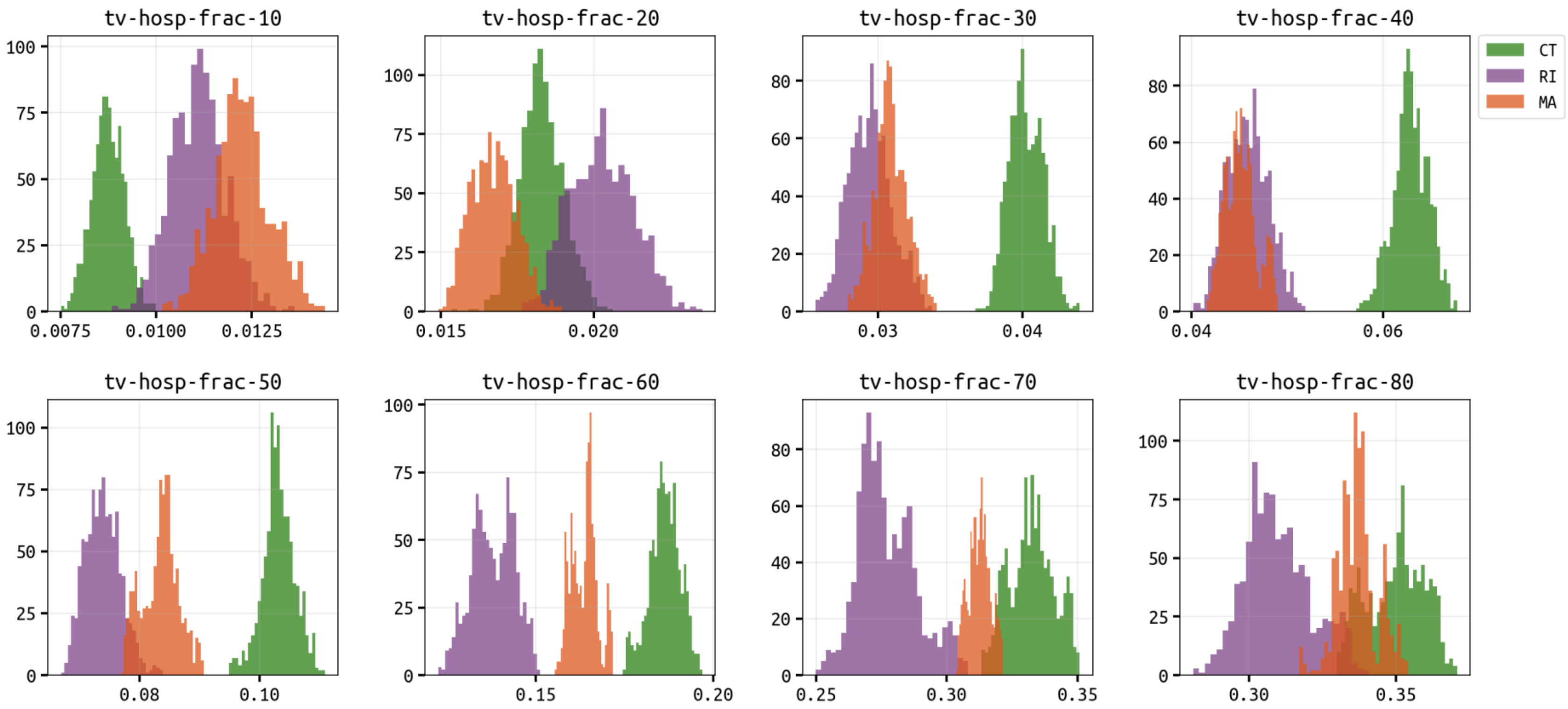

eFigure 5. Panels show posterior distributions for the probability of hospitalization for a symptomatic infection of SARS-CoV-2, by 10-year age band, starting at 10-19, 20-29, through to the 80+ age group. The 0-9 age group is assumed to have the same probability of hospitalization as the 10-19 age group.

© 2022 Tran TNA et al. *JAMA Network Open.*

eFigure 6. Posterior Distributions

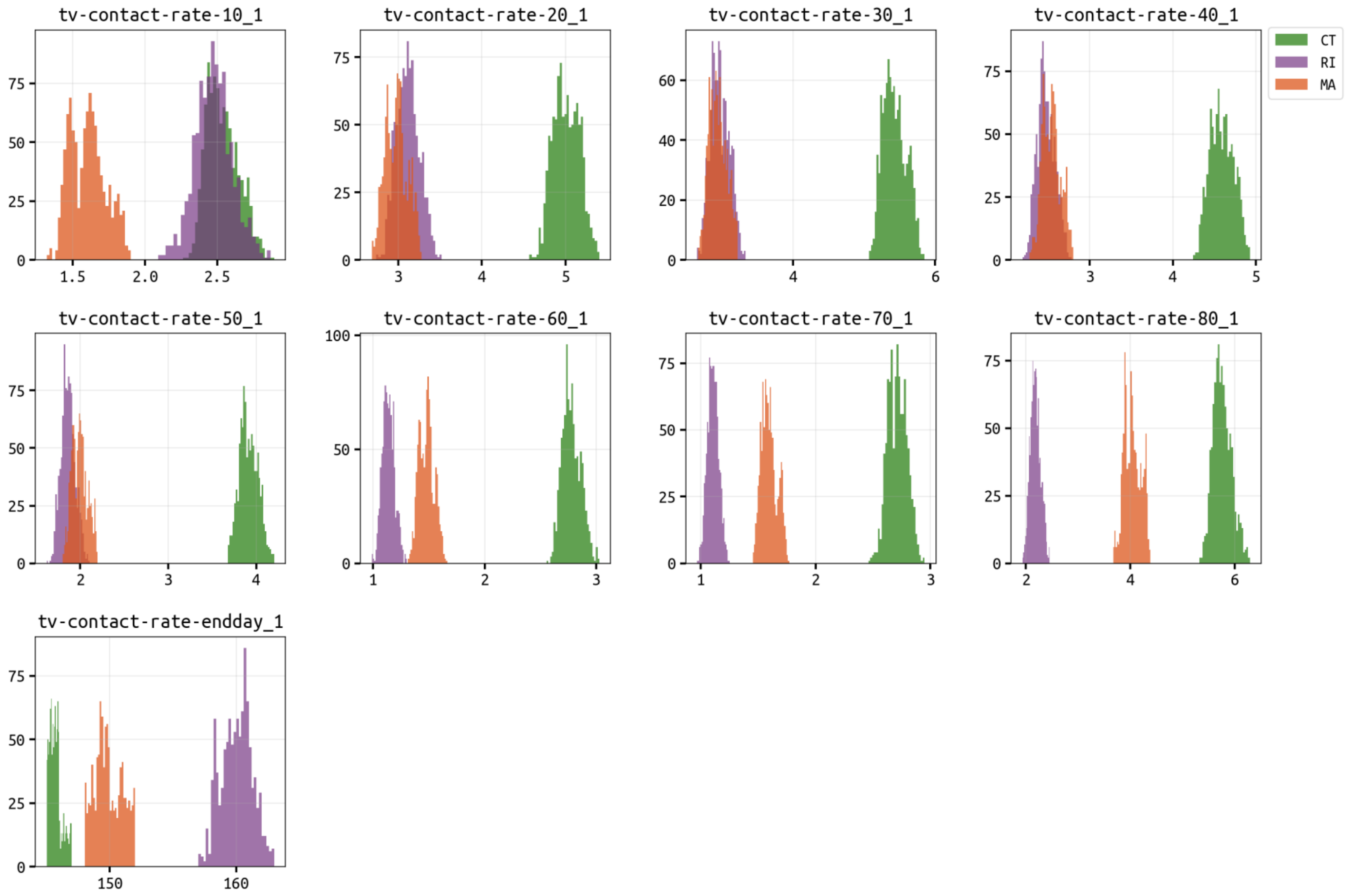

eFigure 6. Panels show posterior distributions for relative "transmission-capable contact rate" for the first period of inference which ends at **tv-contact-rate-endday_1** (posteriors shown in bottom panel; day 150 is May 30 2020). Contact rates are broken down by age group (10-19, 20-29 to 80+) and are all presented as relative to the contact rate of the 0-9 age group.

© 2022 Tran TNA et al. *JAMA Network Open.*

eFigure 7. Posterior Distributions

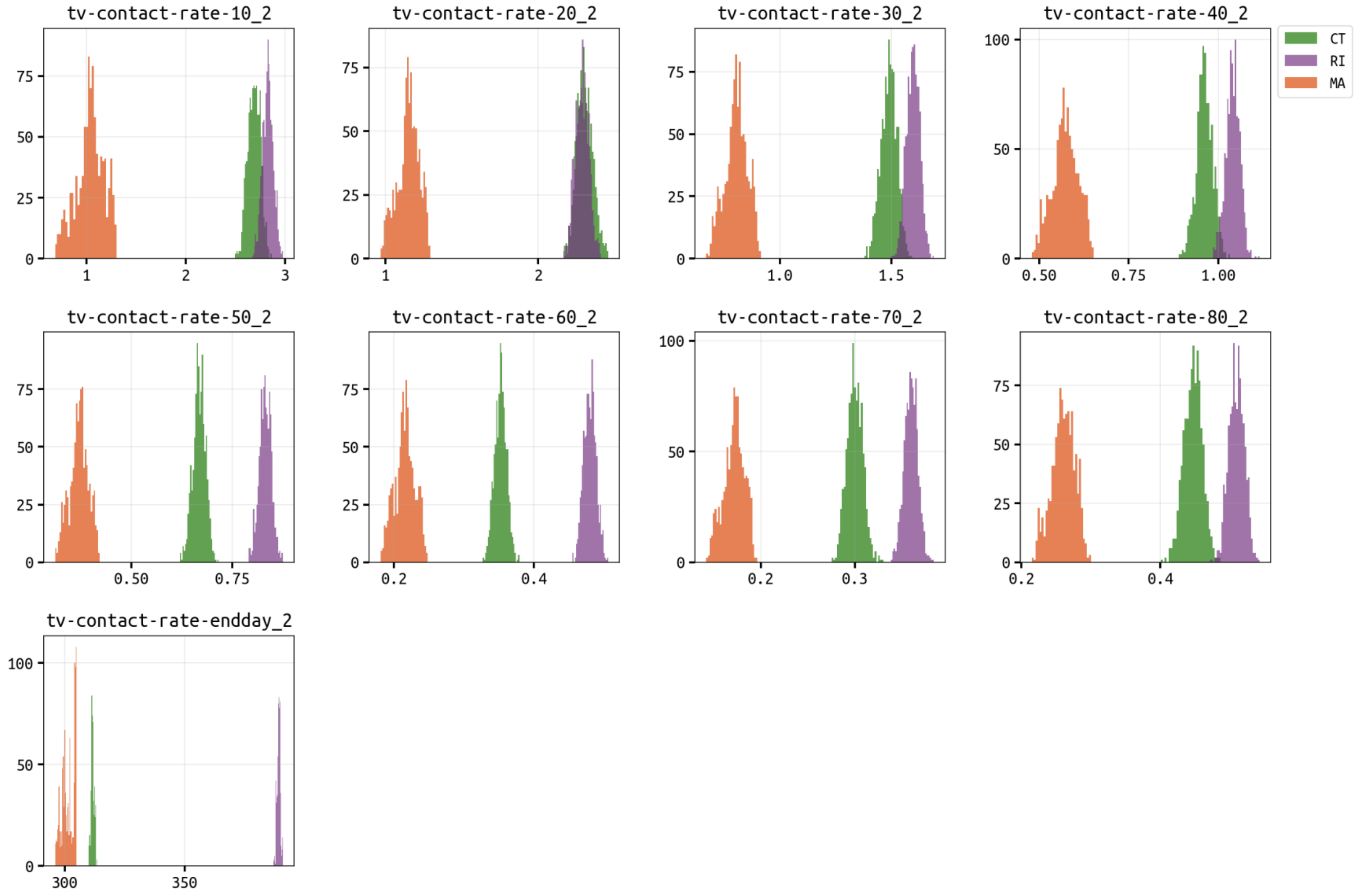

eFigure 7. Panels show posterior distributions for relative "transmission-capable contact rate" for the second period of inference which ends at `tv-contact-rate-endday_2` (posteriors shown in bottom panel; day 300 is Oct 26 2020). Contact rates are broken down by age group (10-19, 20-29 to 80+) and are all presented as relative to the contact rate of the 0-9 age group.

© 2022 Tran TNA et al. *JAMA Network Open.*

eFigure 8. Posterior Distributions

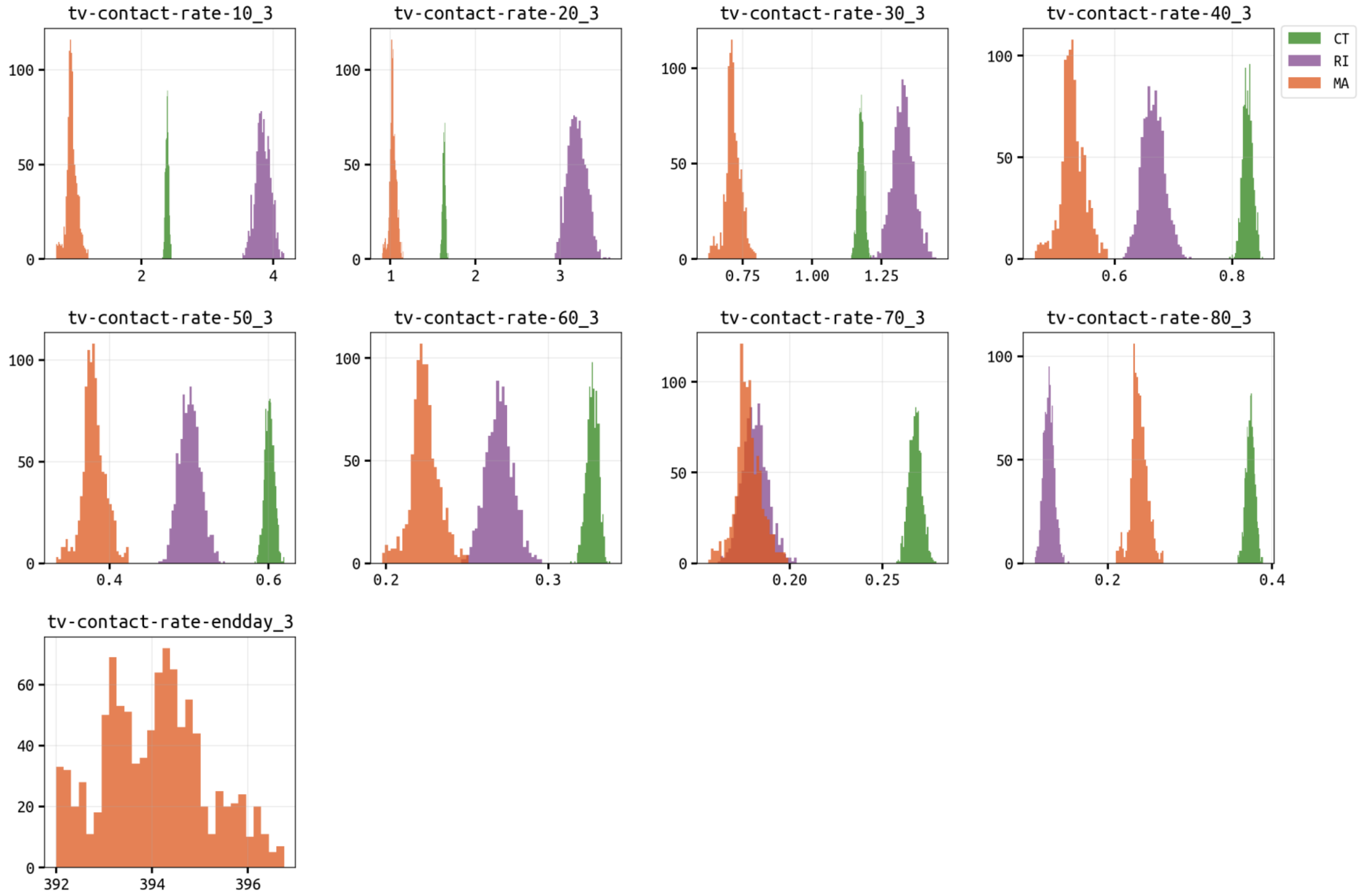

eFigure 8. Panels show posterior distributions for relative "transmission-capable contact rate" for the third period of inference which ends at **tv-contact-rate-endday_3** for Massachusetts (posterior shown in bottom panel; day 394 is Jan 28 2021) or ends on May 31 2021 for Rhode Island and Connecticut. Contact rates are broken down by age group (10-19, 20-29 to 80+) and are all presented as relative to the contact rate of the 0-9 age group.

© 2022 Tran TNA et al. *JAMA Network Open.*

eFigure 9. Posterior Distributions

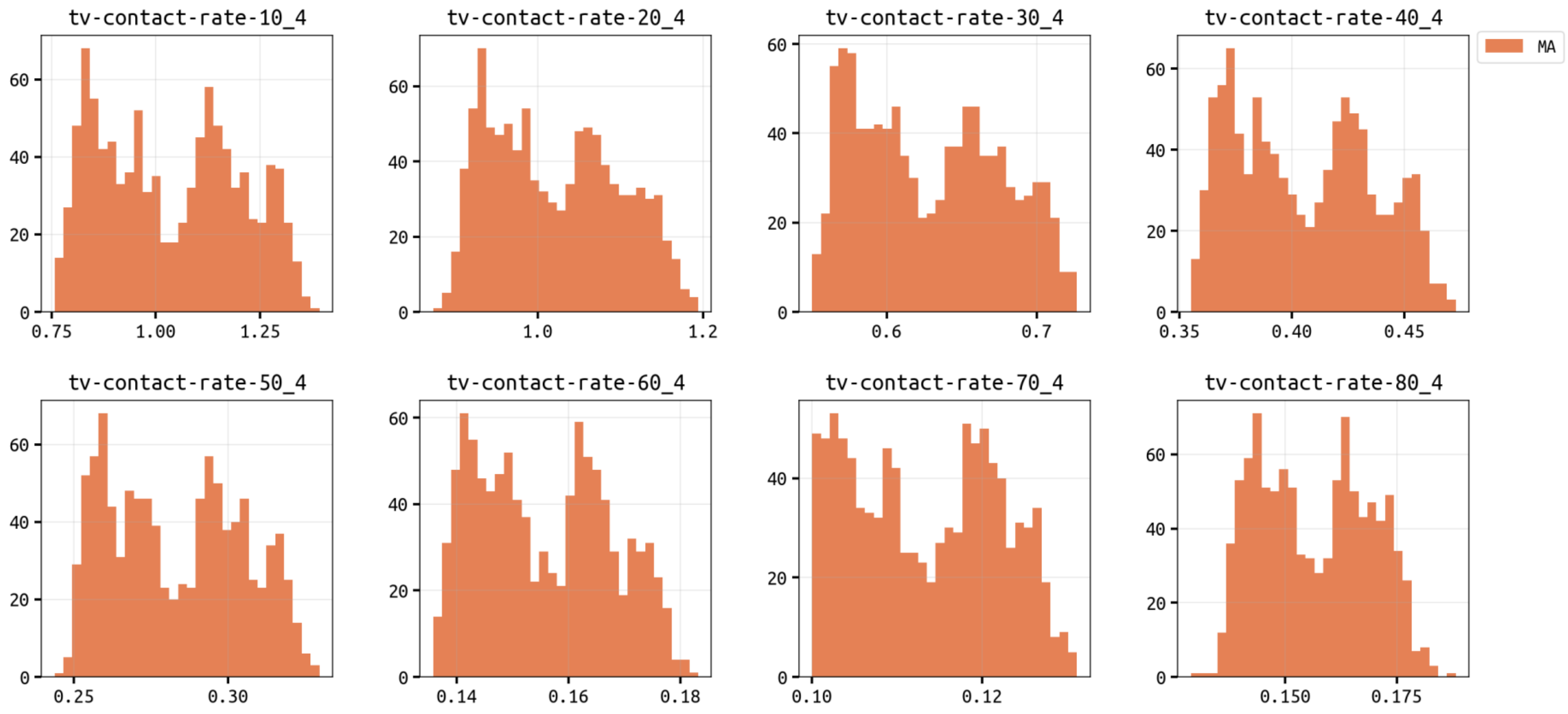

eFigure 9. Panels show posterior distributions for relative "transmission-capable contact rate" for the fourth period of inference which ends was only used for the Massachusetts inference (based on DIC and visual fit). Contact rates are broken down by age group (10-19, 20-29 to 80+) and are all presented as relative to the contact rate of the 0-9 age group.

© 2022 Tran TNA et al. *JAMA Network Open.*

eFigure 10. Alternate Visualization for Hospitalization Probability

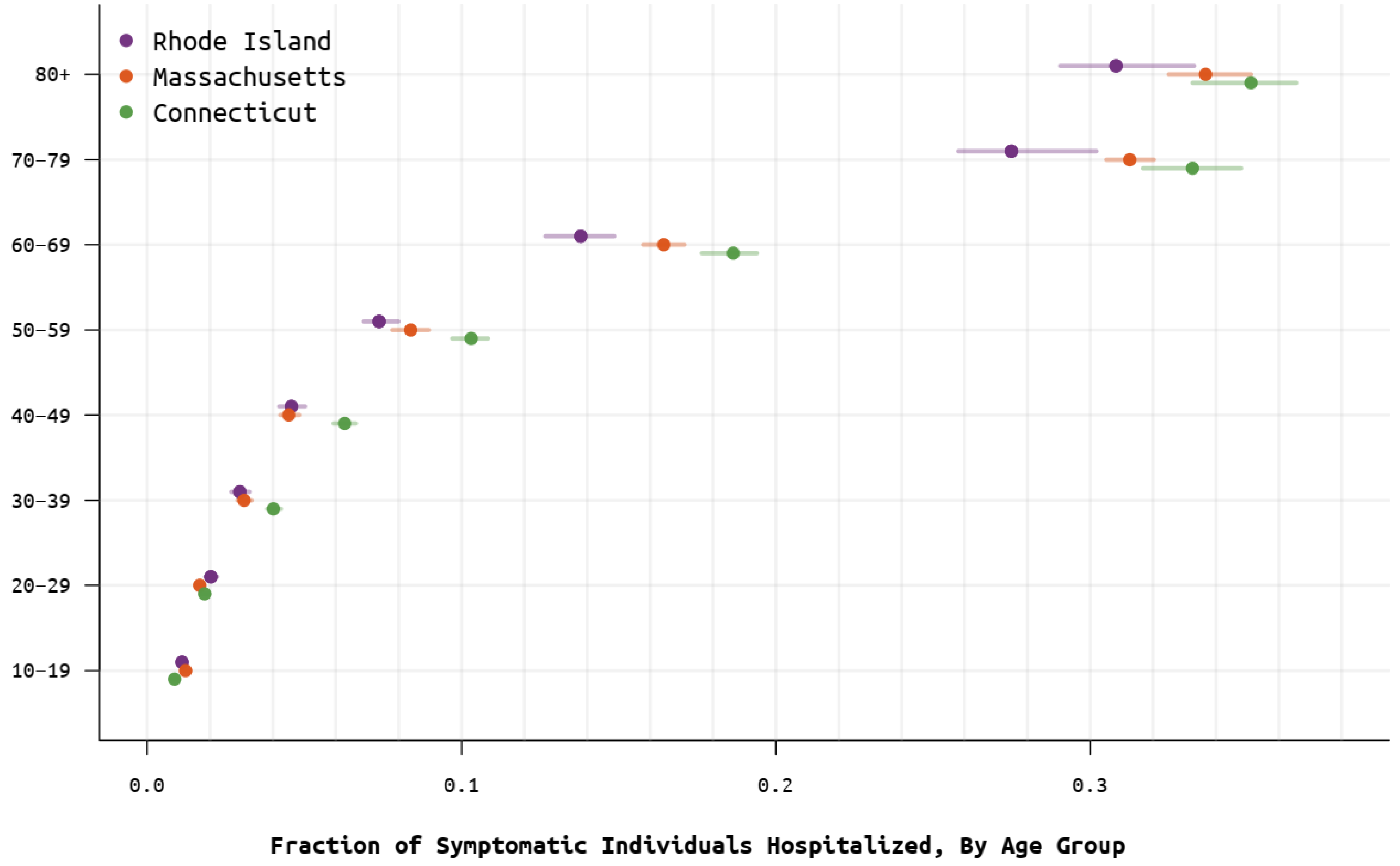

eFigure 10. Age-specific probability (*x*-axis) of hospitalization by age group (*y*-axis). Dots are medians and bars represent 95% credible intervals. Colors represent the three different states. This visualization is simply an alternate view of the posteriors presented in eFigure 5.

© 2022 Tran TNA et al. *JAMA Network Open.*
